# Circulating Donor-Specific Anti-HLA Antibodies Associate With Immune Activation Independent of Kidney Transplant Histopathological Findings

**DOI:** 10.3389/fimmu.2022.818569

**Published:** 2022-02-23

**Authors:** Elisabet Van Loon, Baptiste Lamarthée, Thomas Barba, Sandra Claes, Maarten Coemans, Henriette de Loor, Marie-Paule Emonds, Priyanka Koshy, Dirk Kuypers, Paul Proost, Aleksandar Senev, Ben Sprangers, Claire Tinel, Olivier Thaunat, Amaryllis H. Van Craenenbroeck, Dominique Schols, Maarten Naesens

**Affiliations:** ^1^ Department of Microbiology, Immunology and Transplantation, Nephrology and Kidney Transplantation Research Group, Katholieke Universiteit (KU) Leuven, Leuven, Belgium; ^2^ Department of Nephrology and Kidney Transplantation, University Hospitals Leuven, Leuven, Belgium; ^3^ Department of Transplantation, Nephrology and Clinical Immunology, Edouard Herriot Hospital Lyon, Hospices Civils de Lyon, Lyon, France; ^4^ Department of Microbiology, Immunology and Transplantation, Laboratory of Virology and Chemotherapy, Rega Institute, Katholieke Universiteit (KU) Leuven, Leuven, Belgium; ^5^ Leuven Biostatistics and Statistical Bioinformatics Centre, Department of Public Health and Primary Care, Katholieke Universiteit (KU) Leuven, Leuven, Belgium; ^6^ Histocompatibility and Immunogenetics Laboratory, Red Cross-Flanders, Mechelen, Belgium; ^7^ Department of Imaging and Pathology, Katholieke Universiteit (KU) Leuven, Leuven, Belgium; ^8^ Laboratory of Molecular Immunology, Department of Microbiology, Immunology and Transplantation, Rega Institute for Medical Research, Katholieke Universiteit (KU) Leuven, Leuven, Belgium

**Keywords:** cytokines, chemokines, donor-specific anti-HLA antibodies, allograft rejection, kidney transplantation

## Abstract

Despite the critical role of cytokines in allograft rejection, the relation of peripheral blood cytokine profiles to clinical kidney transplant rejection has not been fully elucidated. We assessed 28 cytokines through multiplex assay in 293 blood samples from kidney transplant recipients at time of graft dysfunction. Unsupervised hierarchical clustering identified a subset of patients with increased pro-inflammatory cytokine levels. This patient subset was hallmarked by a high prevalence (75%) of donor-specific anti-human leukocyte antigen antibodies (HLA-DSA) and histological rejection (70%) and had worse graft survival compared to the group with low cytokine levels (HLA-DSA in 1.7% and rejection in 33.7%). Thirty percent of patients with high pro-inflammatory cytokine levels and HLA-DSA did not have histological rejection. Exploring the cellular origin of these cytokines, we found a corresponding expression in endothelial cells, monocytes, and natural killer cells in single-cell RNASeq data from kidney transplant biopsies. Finally, we confirmed secretion of these cytokines in HLA-DSA-mediated cross talk between endothelial cells, NK cells, and monocytes. In conclusion, blood pro-inflammatory cytokines are increased in kidney transplant patients with HLA-DSA, even in the absence of histology of rejection. These observations challenge the concept that histology is the gold standard for identification of ongoing allo-immune activation after transplantation.

## Introduction

Despite marked improvement in short-term graft outcome through reduced incidence of acute T-cell-mediated rejection (TCMR) with the current immunosuppressive armamentarium, long-term kidney allograft survival remains suboptimal (1, 2). An important cause for this long-term graft failure is acute rejection, especially antibody-mediated rejection (ABMR) (3, 4). ABMR is initiated by donor-specific antibodies (DSA), either to human leukocyte antigens (HLA) or less commonly, but also less easily detectable, to other donor-recipient mismatched antigens (5, 6). Part of the explanation for the impaired graft survival associated with ABMR (7–10) is the lack of proven effective treatments to prevent or treat ABMR (4, 11, 12).

In the search for biomarkers and therapeutic targets for acute rejection, several research groups have suggested to study cytokines, as they play a crucial role in the pathophysiology of rejection (13–31). However, studies focusing on only one or few molecules miss the complexity of the interactions and the interplay between the full landscape of cytokines. Also, details on the specificity of some cytokines for allo-immune processes, different rejection subtypes and associated histological lesions is lacking.

Therefore, we evaluated the complex landscape of 28 cytokines, chemokines, and growth factors in the blood of patients with a broad range of acute rejection types and histological lesions and analyzed the cellular origin of the most relevant cytokines, through publicly available single-cell RNASeq data and through *in vitro* models of HLA-DSA-mediated activation of NK cells, monocytes, and endothelial cells.

## Materials and Methods

### Patient Population

We included all adequate for-cause (“indication”) biopsies performed within the first year after transplantation between August 07, 2012, and July 13, 2016, from consenting patients who received a single kidney transplant at the University Hospitals Leuven, Belgium. All patients gave written informed consent for collection and analysis in the Kidney Transplant Biobank, approved by the local ethical committee (S53364 and S61971). Details on the data collection are described in the **Supplementary Appendix**.

### Histopathology

Histological lesions were semiquantitatively scored according to the Banff consensus (32), as reported previously (33). Diagnosis of the phenotypes of ABMR, TCMR, and borderline changes was established using the Banff 2019 criteria (34). More details are described in the **Supplementary Appendix**.

### Detection of Circulating Anti-HLA Antibodies

The follow-up of anti-HLA antibodies was systematically monitored in one histocompatibility laboratory (HILA—Belgian Red Cross Flanders); details on this assessment were previously published (35), and further details are provided in the **Supplementary Appendix**.

### Cytokine Quantification

Peripheral blood serum samples were analyzed for 28 cytokines, chemokines, and growth factors with Bio-Plex immunoassay using Luminex magnetic beads following the manufacturer’s instructions (Bio-Rad, M50-0KCAF0Y; Temse, Belgium) using a 27-multiplex panel and an additional single-plex one for CXCL9. Details are provided in **Supplementary Table S1** and **Supplementary Appendix**.

### Single-Cell RNA Sequencing Data Analysis

Previously published human single-cell data from two ABMR biopsies and five healthy references corresponding to transplant surveillance biopsies were used. The associated raw counts or matrices were downloaded from the Gene Expression Omnibus (GEO, GSE145927, https://www.ncbi.nlm.nih.gov/geo) (36) and Kidney Precision Medicine Project (https://atlas.kpmp.org/repository). Details on the gene expression analyses are described in the **Supplementary Appendix**. The CellChat R package (37) was used to analyze the cell–cell communication between these cells.

### NK Cell and Monocyte Sorting and Coculture With Endothelial Cells

Peripheral blood mononuclear cells were isolated from the blood of healthy volunteers from the Etablissement Français du Sang (Lyon, France) or from the Belgian Red Cross Flanders by Ficoll gradient centrifugation (Eurobio, Courtaboeuf, France). Cell sorting methods are detailed in **Supplementary Appendix**. Purified NK cells and non-classical monocytes were then cocultured with glomerular endothelial cells. In a first experiment, NK cells were cocultured with the human conditionally immortalized glomerular endothelial cell line (38) (ciGENC; HLA-A2), in combination with either anti-HLA-A2 DSA-containing serum or control human serum. After 4 h of coculture, supernatants were collected. In a second experiment, NK cells and non-classical monocytes were cocultured with primary glomerular endothelial cells (GENC, Cell Systems, USA), after incubation with either anti-HLA-A, -B, -C purified antibody (BD Biosciences, San Jose, CA, USA; cat #560187), or control isotype (BD Biosciences, cat #553447). After 24 h of coculture, supernatants were collected. Details on the two experiments are specified in the **Supplementary Appendix**.

Levels of 27 cytokines, chemokines, and growth factors in the supernatants were assessed using the same 27-multiplex analysis as used for the clinical samples, following the manufacturer’s instructions (Bio-Rad, M50-0KCAF0Y; Bio-Rad, Nazareth, Belgium).

### Statistical Analysis

We report descriptive statistics using mean and standard deviation (or median and interquartile range for skewed distributions) for continuous variables or numbers, and percentages for discrete variables. Protein levels were log10-transformed for analysis. We restricted the Pearson correlation analyses to the first sample per patient, to avoid dependent measurements. Principal component analysis (PCA) and heatmap analysis were done using Euclidean distance to obtain the distance matrix and complete agglomeration method for clustering in the heatmap analysis. In logistic regression analysis, we corrected for repeat sampling by using mixed models with a random intercept. We controlled for multiple testing using the false discovery rate (FDR). Cox regression analyses and Kaplan–Meier survival curves with log-rank testing were used to assess associations with death-censored graft failure. Patients were censored at the time of death with a functioning graft or at the time of the last follow-up date. Time to failure was counted starting at the day of sample. We assessed the differences in protein levels in the *in vitro* experiments using paired T-tests on the log10-transformed protein levels. We used SAS (version 9.4; SAS Institute, Cary, NC), R (version 3.6.3, R core team, 2014, packages corrplot, FactoMineR, heatmap3), and GraphPad Prism (version 8; GraphPad Software, San Diego, CA) for statistical analysis and data presentation. Figures were created with BioRender.com.

## Results

### Blood Cytokine Profiles Associate With HLA-DSA

**Figure 1:**
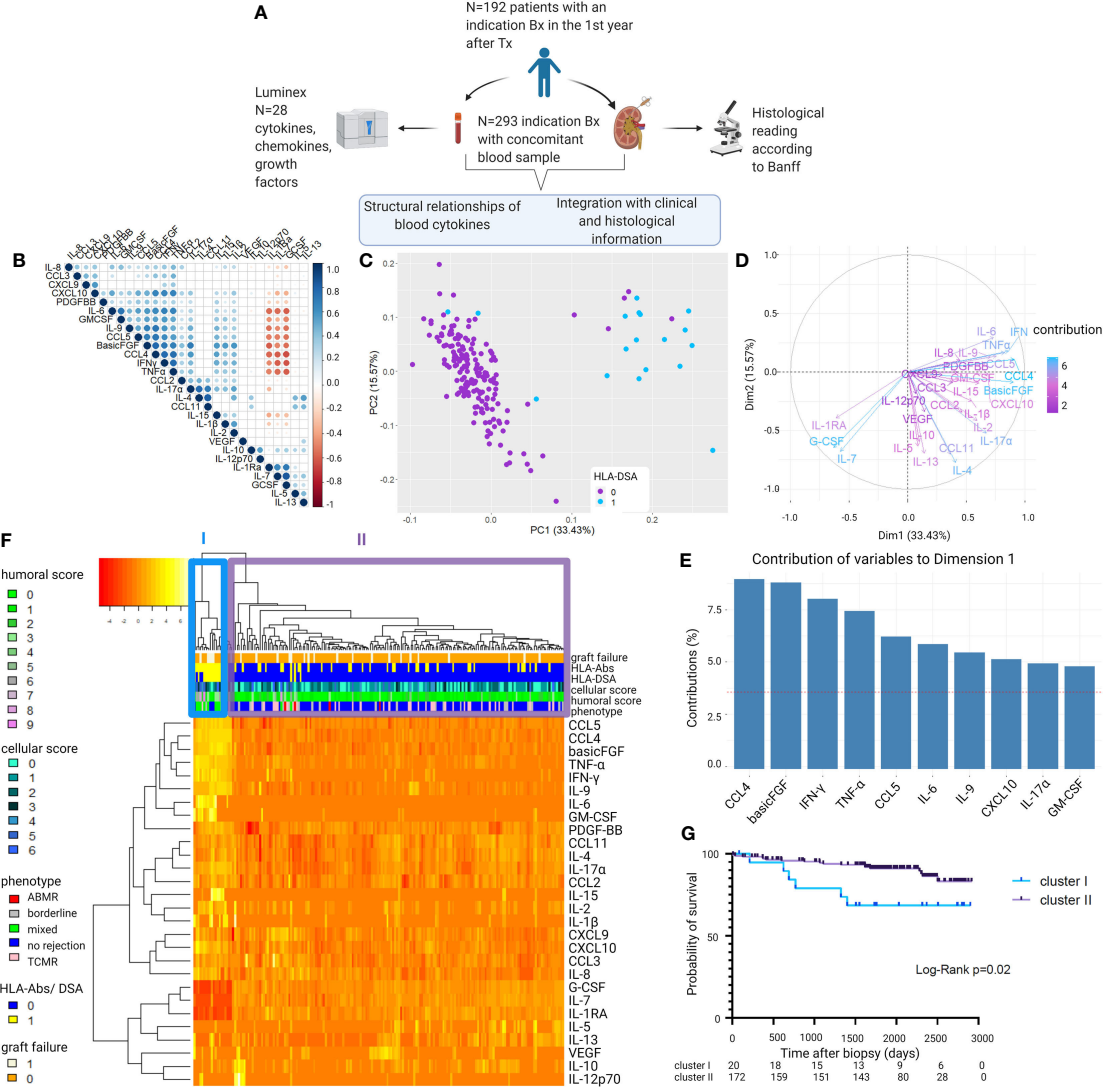
Visualization of the structural relationships between blood protein levels and clinical and histological characteristics. **(A)** Study design. **(B)** Correlation matrix of the 28 proteins using Pearson correlations of the log10-transformed protein levels. Colors indicate correlation coefficient (ρ); dots indicate p-values (only p-values <0.05 are represented with a circle). Proteins are ordered as defined by hierarchical clustering. **(C)** Principal component analysis (PCA) of first biopsies per patient (N = 192) shows two clusters distinct in their 28 blood protein levels. Colors indicate the presence of HLA-DSA. **(D)** Contributions of the 28 cytokines to the principal component analysis. **(E)** Top 10 contributing cytokines to axis 1 of the principal component analysis (PC1). **(F)** Heatmap analysis of histological lesions and blood proteins of first biopsies per patient (N = 192). Reordering of dendrograms based on hierarchical clustering. Two distinct clusters, cluster I (N = 20) and II (N = 172) can be distinguished. **(G)** Kaplan–Meier survival curve illustrating survival probability for cluster I and II, counted from the day of the first biopsy (N = 192). ABMR, antibody-mediated rejection; TCMR, T cell-mediated rejection; HLA-DSA, anti-HLA donor-specific antibodies; HLA-Abs, anti-HLA antibodies. Mixed rejection was defined as ABMR concomitant with TCMR or borderline changes. All protein levels are log10 transformed. Humoral score = sum of glomerulitis; peritubular capillaritis, intimal arteritis and C4d deposition in the peritubular capillaries. Cellular score = sum of tubulitis, interstitial inflammation, and intimal arteritis.

In the first samples of each patient (N = 192; **Figure 1A**, **Table 1** and **Supplementary Table S2**), strong positive correlations were demonstrated among cytokines CXCL10/IP10, PDGF-BB, IL-6, GM-CSF, IL-9, CCL5/RANTES, basic-FGF, CCL4/MIP-1β, TNF-α, IFN-γ, CCL2/MCP-1, and IL-17α, followed by IL-15, IL-1β, and IL-2, and negative correlations with IL-1Ra, IL-7, and G-CSF (**Figure 1B**). PCA based on the 28 blood proteins in first samples revealed two distinct clusters (**Figure 1C**). The pro-inflammatory cytokines IFN-γ, TNF-α, IL-6, IL-9, IL-17α, CXCL10/IP-10, CCL4/MIP1β, CCL5/RANTES, basic-FGF, and GM-CSF (**Figures 1D, E**) were the strongest contributors to differentiate a cluster of 20/192 (10.4%) samples, which was hallmarked by positivity for HLA-DSA (15/20; 75%), whereas the majority of the other cluster were HLA-DSA negative (169/172; 98.3%) (**Figure 1C**). Unsupervised hierarchical clustering confirmed this differentiation in the levels of pro-inflammatory cytokines, corresponding to the HLA-DSA status, and the histological lesions scores (cluster I) (**Figure 1F**). In 14/15 HLA-DSA+ patients in cluster I, HLA-DSA were present at time of the biopsy (12 persistent pretransplant, 1 *de novo*, and 1 both pretransplant and *de novo* HLA-DSA), whereas in 1 patient the HLA-DSA were present pretransplant but had resolved at the first biopsy. In the other five patients (25%) of cluster I, no HLA-DSA was detected at the time of transplantation or at any time post-transplantation. No differences in specificity (class) or strength (MFI) of the HLA-DSA were noted between the HLA-DSA+ samples in cluster I (N = 15 HLA-DSA) vs. cluster II (N = 3 HLA-DSA) (**Supplementary Table S3**). When comparing patients in cluster I vs. II, differences were noted in HLA-DSA, repeat transplantation, induction therapy, and recipient sex, all reflecting the background risk and management of HLA-DSA, but no differences in C-reactive protein or polyomavirus or cytomegalovirus viremia (**Table 2**). When repeating the analyses in all samples (N = 293), including the repeat sample-biopsy pairs, a similar cluster of HLA-DSA+ samples resulted from the PCA and heatmap analyses (**Supplementary Figure S1**). Follow-up samples from patients whose first biopsy was clustered in cluster I clustered together in the pro-inflammatory cluster.

**Table 1:** Sample characteristics of first and all biopsies.

	*First biopsies (N = 192)*	*All biopsies (N = 293)*
	*Mean ± std*	
	*Or median (IQR)*	
	*Or no (%)*	
*Clinical parameters*		
Serum C reactive protein, mg/L	9.0 (1.8–24.7)	5.3 (1.4–18.3)
eGFR, MDRD, mL/min	19.6 ± 13.1	19.8 ± 12.2
Proteinuria, g/g creatinine	0.3 (0.2–1.1)	0.3 (0.2–0.8)
Time after transplantation (days)	11 (7–24)	18 (9–51)
HLA-DSA at time of biopsy	17 (8.9%)	29 (9.9%)
HLA-DSA at time of transplant	17 (8.9%)	34 (11.6%)
*De novo* HLA-DSA	2 (1.0%)	6 (2.1%)
*Histological diagnosis*		
Any rejection	72 (37.5%)	106 (36.2%)
- ABMR (pure)	5 (2.6%)	7 (2.4%)
- TCMR (pure)	35 (18.2%)	54 (18.4%)
- Borderline changes (pure)	22 (11.5%)	26 (8.9%)
- Mixed rejection	10 (5.2%)	19 (6.5%)
*Histological lesion scores*		
Glomerulitis > 0	33 (17.2%)	53 (18.1%)
Peritubular capillaritis > 0*^a^*	19 (9.9%)	37 (12.6%)
Tubulitis >0	128 (66.7%)	200 (68.3%)
Interstitial inflammation >0	51 (26.6%)	73 (24.9%)
Intimal arteritis >0	38 (19.8%)	62 (21.2%)
C4d ptc >1*^a^*	17 (8.9%)	24 (8.2%)
Trombi (yes)	10 (5.2%)	14 (4.8%)
ABMRh (yes)	23 (12.0%)	41 (14.0%)
Arteriolar hyalinosis >1	25 (13.0%)	44 (15.0%)
Vascular intimal thickening >1	40 (20.8%)	70 (23.9%)
Interstitial fibrosis >1	7 (3.7%)	25 (8.5%)
Tubular atrophy >1	4 (2.1%)	11 (3.8%)
Transplant glomerulopathy >0	0	3 (1.0%)
Mesangial matrix expansion>0	10 (5.2%)	19 (6.5%)

^a^Missing data for peritubular capillaritis (N = 5 in first biopsies, and N = 6 in all biopsies), C4d deposition in ptc (N = 6 in first biopsies, N = 9 in all biopsies).

**Table 2:** Comparison of clinical characteristics between cluster I (N = 20) and cluster II (N = 172).

	Cluster I	Cluster II	p-value
Recipient age (years)	52.0 ± 11.7	55.9 ± 11.6	0.16
Donor age (years)	47.2 ± 14.8	54.3 ± 15.2	0.05
Recipient sex (male)	7 (35.0%)	126 (73.3%)	**0.0004**
Donor sex (male)*^a^*	12 (60.0%)	98 (58.0%)	0.86
Cold ischemia time (hours)*^a^*	11.2 ± 5.8	11.9 ± 5.5	0.59
Type of donor			0.55
- Circulatory death	3 (15.0%)	39 (22.7%)	
- Brain death	15 (75.0%)	124 (72.1%)	
- Living	2 (10.0%)	9 (5.2%)	
Repeat transplantation (yes)	6 (30.0%)	17 (9.9%)	**0.009**
Immunosuppressive regimen (TAC-MMF-CS)	20 (100%)	155 (90.1%)	0.14
Induction therapy	12 (60.0%)	54 (31.4%)	**0.01**
Polyoma viremia (positive)*^a^*	0 (0%)	15 (8.7%)	0.20
CMV viremia (positive)*^a^*	0 (0%)	9 (5.2%)	0.34
Serum C reactive protein, mg/L	8.7 (1.8–25.3)	10.3 (2.9–21.1)	0.66
eGFR, MDRD, mL/min	17.7 ± 11.9	19.8 ± 13.2	0.49
Proteinuria, g/g creatinine	0.3 (0.2–1.1)	0.5 (0.2–1.2)	0.30
Time after transplantation (days)	11 (7–24)	10 (7–23)	0.53
HLA-DSA at time of biopsy	14 (70.0%)	3 (1.7%)	**<0.0001**
HLA-DSA at time of transplant	14 (70.0%)	3 (1.7%)	**<0.0001**
*De novo* HLA-DSA	2 (10.0%)	0 (0%)	**<0.0001**
Any rejection	14 (70.0%)	58 (33.7%)	**<0.0001**
- Pure ABMR	1 (5.0%)	4 (2.3%)	
- Pure TCMR	2 (10.0%)	33 (19.2%)	
- Pure borderline changes	3 (15.0%)	19 (11.1%)	
- Mixed rejection	8 (40.0%)	2 (1.2%)	
Acute histological lesions			
- Glomerulitis > 0	10 (50.0%)	23 (13.4%)	**<0.0001**
- Peritubular capillaritis > 0*^a^*	6 (30.0%)	13 (7.8%)	**0.002**
- Tubulitis >0	16 (80.0%)	112 (65.1%)	0.18
- Interstitial inflammation >0	11 (55.0%)	40 (23.3%)	**0.002**
- Intimal arteritis >0	7 (35.0%)	31 (18.0%)	0.07
- C4d ptc >1*^a^*	8 (40.0%)	9 (5.4%)	**<0.0001**
- Thrombi (yes)	4 (20.0%)	6 (3.5%)	**0.002**
- ABMRh (yes)	9 (45.0%)	14 (8.1%)	**<0.0001**

^a^Missing data: donor sex N = 3; cold ischemia time N = 3; peritubular capillaritis N = 5; C4d deposition ptc N = 6; polyoma viremia N = 6; CMV viremia N = 40.

TAC, tacrolimus; MMF, mycophenolate mofetil; CS, corticosteroids.

Significant differences are indicated in bold.

**Figure 2:**
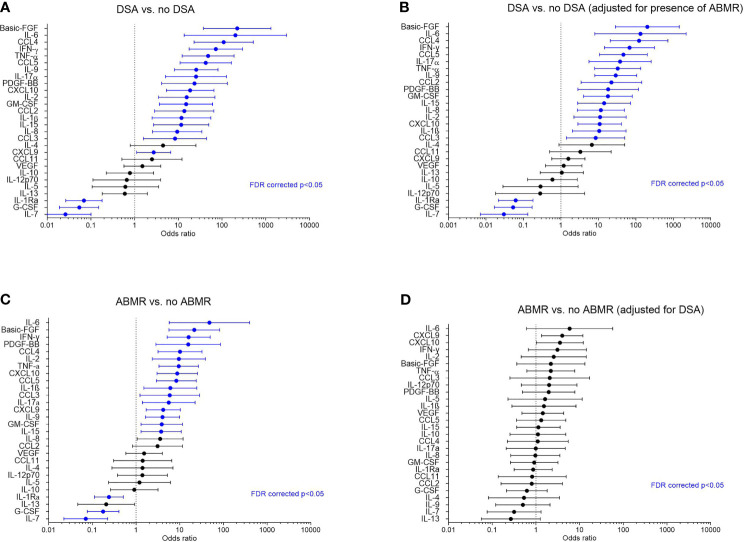
Associations of the cytokines with HLA-DSA and antibody-mediated rejection in all samples (N = 293). **(A)** Forest plots of odds ratios for association of the cytokines with HLA-DSA (N = 38 vs. 255), **(B)** with HLA-DSA when adjusted for presence of ABMR, **(C)** with ABMR vs. no ABMR (N = 26 vs. N =267), and **(D)** with ABMR when adjusted for presence of HLA-DSA. Blue color indicates associations that were significant after false discovery rate correction (FDR p-value <0.05). ABMR, antibody-mediated rejection incl. mixed rejection; HLA-DSA, anti-HLA donor-specific antibodies; FDR, false discovery rate.

As the clustering approach indicated a strong association between the global protein levels and HLA-DSA, we next evaluated which proteins individually associated with HLA-DSA using logistic mixed models. All proteins except IL-4, IL-5, IL-10, IL-12p70, IL-13, CCL11/Eotaxin, and VEGF significantly associated with HLA-DSA (**Figure 2A**). A high number of samples had levels below the detection limit in both clusters for IL-5, IL-10, IL-12p70, IL-13, and VEGF (**Supplementary Table S1** and **Supplementary Figure S2**).

### Cytokine Profiles in Relation to Histological Inflammation

Next to the association with HLA-DSA, also rejection associated with the cytokine clusters (**Table 2**). Rejection was present in the concomitant biopsy in 14/20 (70%) patients of cluster I vs. 58/172 (33.7%) of cluster II (p < 0.0001). ABMR (including mixed rejection) was present in 9/20 (45%) cases in cluster I vs. 6/172 (3.5%) cases in cluster II (p < 0.0001). Glomerulitis, peritubular capillaritis, thrombi, C4d deposition in the peritubular capillaries, and interstitial inflammation scores differed between the clusters (**Table 2**). In non-rejecting samples in cluster I (N = 6/20), the biopsies showed no inflammatory lesions, except for isolated tubulitis in three of these biopsies.

When considering all biopsies, significant associations were found between cytokines and diagnosis of ABMR (N = 26) vs. no ABMR (N = 267) (**Figure 2C** and **Supplementary Figure S3**). However, after adjustment for HLA-DSA, none of the cytokines remained significantly associated with ABMR (**Figure 2D**). Conversely, the associations between HLA-DSA and the individual proteins remained largely unaltered after adjustment for ABMR (**Figure 2B**). This illustrates that HLA-DSA status is a stronger determinant of the cytokine profiles than ABMR histology. Compared to ABMR, fewer cytokines associated with TCMR (N = 71) vs. no TCMR (N = 222), and none associated with borderline changes vs. no rejection (**Supplementary Figure S3**). When considering pure TCMR (N = 54) vs. no TCMR (N = 223), none of the cytokines remained associated after FDR correction (**Supplementary Figure S3**). These findings were corroborated by the associations between protein levels and inflammatory histological lesions. Basic-FGF, IL-1β, IL-9, IL-2, IL-17α, CCL4/MIP1β, CCL5/RANTES, CXCL9/MIG, IFN-γ, and TNF-α positively and IL-7 negatively associated with microvascular inflammation, the hallmark of ABMR (**Supplementary Figure S4**). Proteins with a positive association with tubulo-interstitial inflammation, the hallmark of TCMR, were CXCL9/MIG and CXCL10/IP10 (**Supplementary Figure S4**). When considering intimal arteritis, which can occur both in ABMR and in TCMR, upregulated proteins were IL-6, CXCL8/IL-8, CXCL9/MIG, CXCL10/IP10, IFN-γ, and TNF-α; downregulated proteins were IL-7, IL-13, and G-CSF (**Supplementary Figure S4**).

### Association of Blood Cytokines With Future Rejection and Graft Survival

Of the 14 patients in cluster I with rejection in the first biopsy, 12 (85.7%) had recurring rejections in the follow-up biopsies. Of the 6 patients in cluster I without rejection in the first biopsy, 2 patients developed transplant glomerulopathy in follow-up biopsies without previous acute rejections detected on pathology; 2 patients developed rejection (one TCMR, one borderline); and 2 never developed rejection. In total, 25/192 patients experienced death-censored graft failure during a mean follow-up time of 5.1 ( ± 1.9) years. Graft failure rate differed between cluster I and cluster II (univariable hazard ratio [HR] 2.81, 95% CI 1.12–7.06, p = 0.03) (**Figure 1G**). The association of cluster I with graft failure was independent of recipient sex, repeat transplantation, induction therapy, and ABMR_h_ (adjusted HR 3.31, 95% CI 1.09–10.03, p = 0.03).

### Cellular Origin of the Cytokine Profiles: Single-Cell RNA Sequencing of Kidney Transplant Biopsies

**Figure 3:**
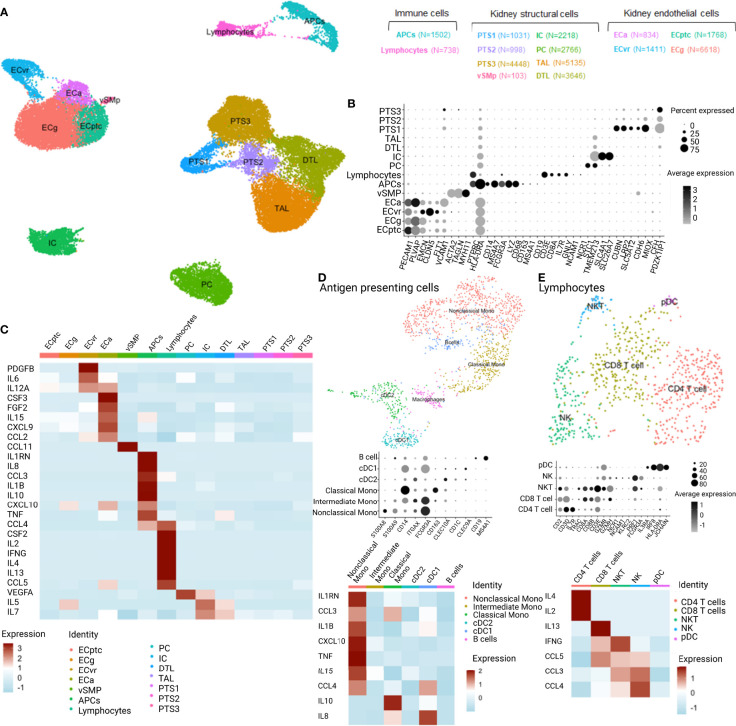
Single-cell RNASeq analysis of the expression distribution of the cytokines of interest across the different cell types distinguished in 7 kidney biopsies analyzed with scRNASeq (2 ABMR, 5 transplant surveillance biopsies). APCs,antigen-presenting cells; PTS, proximal tubular cells; vSMp, vascular smooth muscle and pericytes; IC, intercalated cells; PC, principal cells; TAL, thick ascending loop of Henle; DTL, descending thin limb; ECa, activated endothelial cells; ECvr, endothelial cells of vasa recta; ECptc, endothelial cells of peritubular capillaries and ECg, endothelial cells of glomerulus; cDC, classical dendritic cells; pDC, plasmacytoid dendritic cells; NK, natural killer cells; NKT, natural killer T cells. **(A)** UMAP dimensionality reduction of the different cell types clusters and their relation. **(B)** Dotplot of the identified cell type cluster based on specific marker features. **(C)** Heatmap of the cytokine profiles from the clinical experiment and corresponding expression in the cell types of 7 kidney biopsies. **(D)** UMAP, dotplot, and heatmap of the antigen-presenting cell cluster and cytokine expression. **(E)** UMAP, dotplot, and heatmap of the lymphocyte cluster and cytokine expression.

Based on the observed association between cytokines and HLA-DSA, we hypothesized that these cytokines originated from the transplanted kidneys as the allograft is the site where the DSA-mediated injury occurs. To determine the cellular origin of the cytokine profiles in the allograft, we used publicly available single-cell data from kidney biopsies. Briefly, scRNASeq was performed on two renal allograft biopsies with DSA-mediated injury, namely, ABMR (one with class I and II anti-HLA-DSA (B8, DQA1) and one with class II HLA-DSA [DR1; DR53 and DQ2 (36)], and five reference biopsies. After quality control and filtering (**Supplementary Figure S5**), 33,216 cells were detected and clustering revealed 14 clusters corresponding to the main renal cell subtypes but also infiltrating immune cells (**Figures 3A, B**). After subclustering using well-established markers (39), we evaluated the expression of genes corresponding to the cytokines across the kidney structural cells and infiltrated immune cells (**Figures 3A, B**). The cytokines of interest were expressed by activated endothelial cells (ECa), antigen-presenting cells (APCs), and lymphocytes. More specifically, ECa expressed *CSF3*, *FGF2*, *IL15*, *CXCL9*, and *CCL2*. APCs expressed *IL1RN*, *IL8*, *CCL3*, *IL1B*, *CXCL10*, *IL10, TNF*, and *CCL3* whereas lymphocytes expressed *CCL4, CSF2, IL2, IFNG, IL4, IL13*, and *CCL5* (**Figure 3C**). Of note, *IL9* and *IL17A* were not detected in this dataset. Given the heterogeneity of APC and lymphocyte populations, we subclustered these two populations. In APCs, we identified CD19+MS4A1+ B cells, the three main monocytic populations—classical CD14+CD16-, intermediate CD14+CD16+, and non-classical CD14-CD16+ monocytes—and two dendritic cell (DC) populations (**Figure 3D**). Non-classical monocytes expressed the most relevant cytokines with *IL1RN*, *CCL3*, *IL1B*, *CXCL10, TNF, IL15*, and *CCL4*. In lymphocytes, we detected CD4 T cells, CD8 T cells, NKT cells, NK cells, and plasmacytoid DC, whereas CD4 and CD8 T cells expressed *IL4*, *IL2*, and *IL13*, NK and NKT cells expressed *IFNG*, *CCL3*, *CCL4*, and *CCL5* (**Figure 3E**).

### Cell–Cell Communication

**Figure 4:**
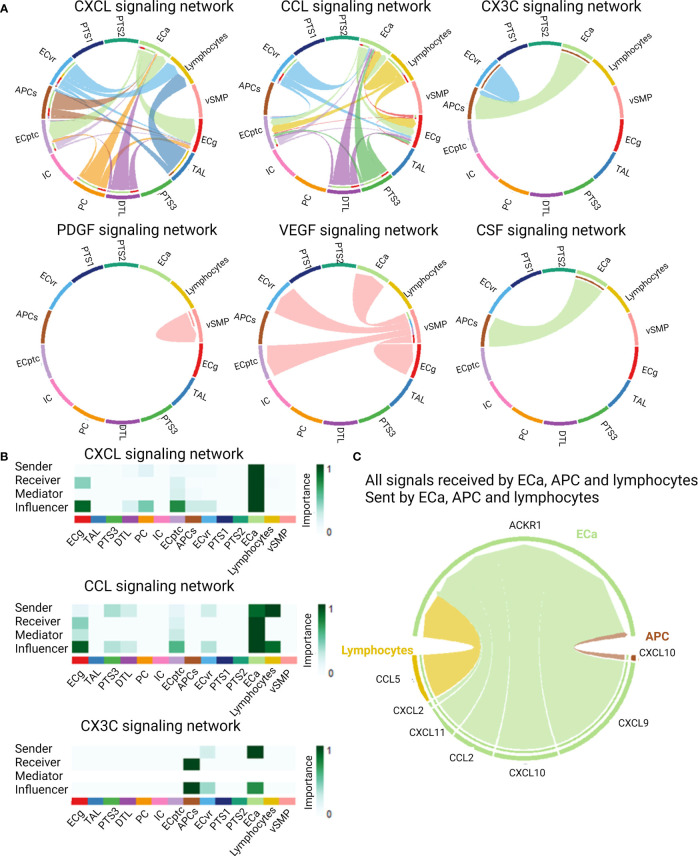
Cell–cell communications derived from scRNASeq data of kidney allograft biopsies. **(A)** Cytokine and chemokine signaling pathways that were significant communication pathways in the kidney allograft cells. **(B)** Most important senders, receivers, mediators, and influencers of the CXCL, CCL, and CX3C signaling network. **(C)** Circos plot demonstrating all signals received by activated endothelial cells (ECa), sent by activated endothelial cells, antigen-presenting cells (APCs), and lymphocytes, illustrating the ligand–receptor pairs.

As cytokines and chemokines are the mediators of cell–cell communication, we used the CellChat package to infer the intercellular communication from the scRNASeq data. Twenty signaling pathways showed significant communication between the allograft cells. Among these were the CXCL, CCL, CX3C, PDGF, VEGF, and CSF signaling pathways (**Figure 4A**). ECa were important senders but also receivers of CXCL and CCL signaling (**Figure 4B**). Lymphocytes were important senders of CCL signaling, whereas APCs were receiving CX3C and CSF signals from ECa in these biopsies. When looking at all signals received by ECa, sent by ECa, APCs, and lymphocytes, the atypical chemokine receptor (ACKR1), also known as DARC, was the identified receptor for all ligand–receptor pairs (**Figure 4C**).

### Cytokine Secretion in Antibody-Mediated NK Cell and Monocyte Activation

**Figure 5:**
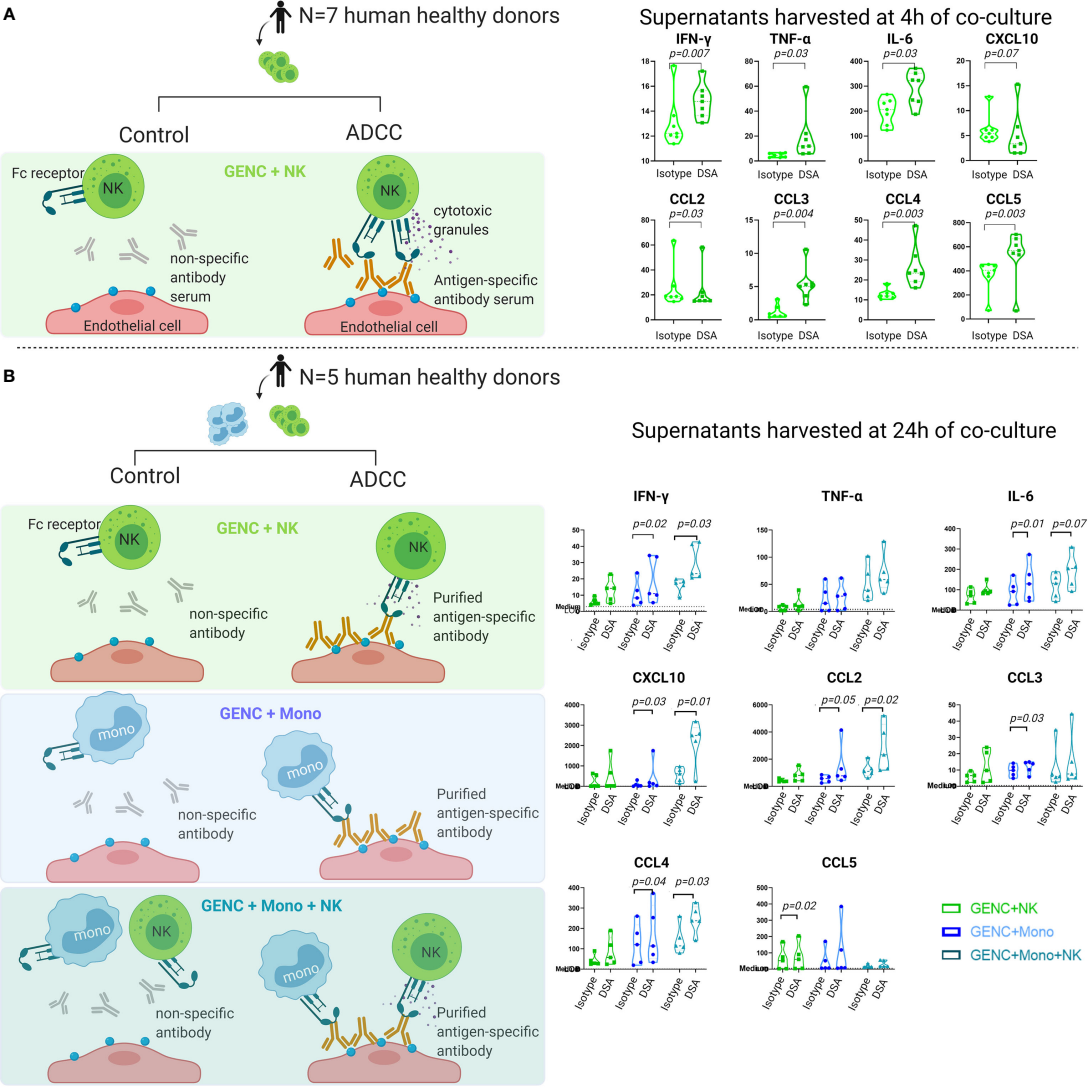
Cytokine profiles in supernatants of NK cells and/or monocytes cocultured with HLA class I antibody-activated glomerular endothelial cells or isotype antibodies. **(A)** NK cell experiment using NK cells from 7 human donors, cocultured with human antibody serum (anti-HLA-A2-containing serum or control pooled serum that was negative for anti-HLA A2) and ciGENC (immortalized glomerular endothelial cells), harvested after 4 h. **(B)** NK cell and monocyte experiment using NK cells and CD16+ monocytes from 5 human donors, cocultured with murine antibody (anti-HLA-A, -B, -C, or control isotype) and GENC (primary glomerular endothelial cells, Cell Systems, USA); harvesting of supernatants was done after 24 h of coculture. Violin plots of the cytokine concentrations (pg/mL) of the significantly differently expressed cytokines in both conditions, as assessed by a paired parametric T-test of the log10 transformed concentrations.

Given that the most clinically relevant cytokines corresponded to expressed genes on activated glomerular endothelial cells, non-classical monocytes, and NK cells, we next assessed the cytokine production by these cells. Indeed, endothelial injury is the hallmark of DSA-mediated injury in kidney allografts and both NK cells and monocytes are key players in ABMR (40–45). Both NK cells and non-classical monocytes express the Fcγ receptor, through which antibody-dependent cellular cytotoxicity can be induced against a target cell, i.e., the endothelial cell in the setting of ABMR. We tested the specificity of the studied proteins for these cell types in an *in vitro* model of antibody-mediated NK cell and/or monocyte activation in interaction with glomerular endothelial cells. After 4 h of coculture with conditionally immortalized glomerular endothelial cells (ciGENC), IFN-γ, TNF-α, IL-6, IL-8, IL-9, IL-15, CCL2/MCP-1, CCL3/MIP1α, CCL4/MIP1β, CCL5/RANTES, CCL11/Eotaxin, and basic-FGF were significantly higher in the supernatants of the antibody-mediated NK cell activation condition, compared to the control condition (**Figure 5A** and **Supplementary Figure S6**). After 24 h of coculture with primary GENC, IFN-γ, IL-4, IL-6, CXCL8/IL-8, CXCL10/IP10, CCL2/MCP-1, CCL3/MIP1α, CCL4/MIP1β, and basic-FGF were significantly higher in the supernatants of the antibody-mediated monocyte activation condition, compared to control (**Figure 5B** and **Supplementary Figure S7**). After 24 h of coculture of NK cells, non-classical monocytes and GENC, IFN-γ, IL-5, CXCL8/IL-8, IL-9, IL-15, CXCL10/IP10, CCL2/MCP-1, CCL4/MIP1β, and GM-CSF were significantly higher in the NK cell and monocyte activation condition, compared to control, suggesting a synergistic effect between these two cell types (**Figure 5B** and **Supplementary Figure S7**). When comparing *in vitro* models of GENC alone, with an HLA class I-specific antibody vs. non-specific isotype antibody, no significant cytokine production was noted (**Supplementary Figure S8**). The cell–cell contact of immune cells with GENC was required for cytokine production, illustrated by the higher cytokine levels in cocultures of immune cells with GENC compared to immune cells alone (**Supplementary Figure S9**).

## Discussion

In this representative cohort of consecutive samples from kidney transplant recipients at time of allograft dysfunction, we demonstrate that pro-inflammatory cytokines in the peripheral blood associate with presence of HLA-DSA and impaired kidney transplant survival. These pro-inflammatory blood signals associate with HLA-DSA, rather than with rejection phenotypes as identified in kidney transplant biopsies. This illustrates that HLA-DSA can lead to immune activation that is not always reflected in histology of the concomitant kidney transplant biopsies. Single-cell data analysis demonstrated that the most contributing cytokines to the clustering of the clinical samples corresponded to genes expressed on activated glomerular endothelial cells, non-classical monocytes, and NK cells. Subsequent *in vitro* models confirmed that the cytokines observed in patients’ serum can be induced by HLA-DSA-mediated cross talk between glomerular endothelial cells, NK cells, and monocytes.

To our knowledge, this is the first study demonstrating the strong association of pro-inflammatory blood cytokine profiles with presence of HLA-DSA. One previous study found a panel of 6 pro-inflammatory cytokines (TNF-α, IFN-γ, IL-1β, IL-6, IL-8/CXCL8, and IL-17) to be associated with angiotensin II type 1 receptor antibodies, a non-HLA antibody, but not with presence of HLA-DSA (13). Most of the cytokines that associated with HLA-DSA in our study are known as potent mediators of the alloimmune responses and associated injury. The function of cytokines and chemokines is characterized by pleiotropism, redundancy, synergism, and antagonism (46), clearly illustrated by the strong correlations we observed between the proteins. The signals that stand out primarily reflect IFN-γ-related inflammation including CXCR3 and CCR5 signaling. Levels of CXCL9/MIG and CXCL10/IP10, ligands to CXCR3, and levels of the MIP-1 family proteins (especially CCL4/MIP-1β and CCL5/RANTES), ligands to CCR5, are strongly associated with diagnosis of presence of HLA-DSA. The importance of these receptors in rejection has been demonstrated in literature, as well as their potential as therapeutic targets (16, 17, 27, 47–52). The presence of both CXCR3 and CCR5 appears to identify subsets of NK cells and T cells in blood with a strong predilection for homing to inflammatory sites (53, 54), and CXCL9/MIG and CXCL10/IP10 have been well characterized in kidney allograft rejection (20, 55).

Next, the scRNASeq analysis on kidney transplant biopsies suggested that, although the different cytokines are highly collinear, they do not originate from the same cells. CXCL9/MIG is mainly expressed by activated endothelial cells, whereas CXCL10/IP10 is expressed by both activated endothelial cells and CD16+ monocytes. These results are in line with reports showing that CD16+ non-classical monocytes present an increased capacity for antibody-dependent cellular cytotoxicity (56). Other relevant identified cytokines are the pro-inflammatory cytokines IL-6, IL-8, IL-9, and IL-17α.

As the scRNASeq analysis suggested expression of the relevant pro-inflammatory cytokine profiles in kidney endothelial cells, monocytes, and NK cells, we next performed *in vitro* studies to evaluate whether these cytokines were indeed produced by these cells upon the presence of HLA-DSA. The strongest increases in cytokine levels were observed when combining both NK cells and monocytes with the glomerular endothelial cells, suggesting a synergistic effect in their cytokine secretion. These observations suggest that cytokine production in the presence of HLA-DSA is FcyR-mediated; however, they do not provide evidence of causality. Future experiments using FcyR blocking or using only the F(Ab′)2 fragments are needed to prove causality. In a previous study by Wei et al. (57) studying monocytes and DSA-mediated injury of endothelial cells, using F(ab′)2 alone induced only upregulation of the antigens on the endothelial cells, leading to increased tethering and adhesion of monocytes. Only in the presence of the Fc region was the extra signal for activation of the monocytes provided, inducing cytokine secretion, suggesting that FcyR-mediated signaling is essential for the cytokine production in DSA-mediated injury.

Since the endothelial cells present in the *in vitro* conditions could contribute to the cytokine profiles observed in these supernatants (58, 59), supernatants of glomerular endothelial cells alone in the presence of a specific anti-HLA antibody vs. isotype antibody were also analyzed. No significant differences in cytokine secretion were noted between the two conditions. Together with the single-cell data, this suggests that endothelial cells express cytokines, but production is only observed when interacting with immune cells. For this interaction with immune cells through chemokines, the ACKR1 receptor, or DARC, was identified as a common receptor on activated endothelial cells for multiple incoming chemokine signals of the CXCL and CCL families. This receptor is known to regulate the dynamics of inflammatory chemokine presentation on vascular endothelial cells and chemokine concentrations in plasma (60). The importance of this receptor in ABMR has been reported previously (28, 61, 62).

Although older studies did demonstrate that HLA class I antibodies can directly trigger cytokine secretion from endothelial cells (15, 63), our data are in accordance with the study of Wei et al., where HLA class I antibodies did not induce cytokine production directly from endothelial cells derived from aortic rings, but cell–cell contact of monocytes with antibody-activated endothelial cells was required for cytokine production (57). The observation that this is also true for glomerular endothelial cells used in our experiments, which are more relevant for kidney microvascular injury, is novel. The discrepancy with previous studies (15, 63) could be explained by differences in vascular origin of the endothelial cells or in the type/concentration of the HLA antibodies.

Our present work challenges the vision that kidney transplant histology is the gold standard for identification of ongoing alloimmune processes. The immune activation detectable in the peripheral blood of patients with HLA-DSA could be an explanation for the bad outcome observed in patients with HLA-DSA, even in the absence of histological lesions in the biopsy (35). This could be related to the observer dependency of kidney transplant biopsy reading and the risk for sampling error, in addition to the potential that classic light microscopy is not sensitive enough for detecting kidney transplant inflammation. As illustrated previously (64), increased intrarenal rejection-associated gene transcripts can be observed in biopsy samples of DSA-positive/ABMR-negative patients, potentially proceeding to later overt histological injury (65). Given that the pathological evaluation of biopsies is observer-dependent and subject to sampling error, inflammatory lesions could be missed or remain below the thresholds of the Banff classification, yet be reflected by inflammatory changes detectable in the graft/circulation. Markers for inflammatory changes in the graft below the threshold of the Banff classification have been suggested before, by studying intragraft transcriptional changes (“molecular microscope”) (66) or donor-derived cell-free DNA (67) as markers for severity of graft injury to complement the histological assessment using Banff classification. Our study illustrates that also blood inflammatory cytokine profiles could help in objectifying deleterious inflammation in patients at high risk of rejection but with negative histology. This is further supported by our finding that patients with HLA-DSA and enhanced pro-inflammatory signals had worse outcome than HLA-DSA-positive patients without pro-inflammatory signals in the blood. All of this seems to be in contrast to the study of Parajuli et al., who did not observe different outcomes in patients with and without HLA-DSA if the index biopsy was negative for histological inflammation (68). This previous study was however focused on first biopsies in the absence of rejection, the study design is not comparable to our strategy with a relatively short follow-up time, and the interpretation of these data could be hampered by inherent selection bias.

There are some limitations to this study. The single-center nature of our study population, consisting largely of white Europeans with low to intermediate immunologic risk, treated mainly with a tacrolimus-based regimen, limits the generalizability of these findings to other and higher-risk populations and warrants external validation. Second, this study consisted of biopsies performed at time of graft dysfunction with possible confounders and inherently excluding cases with subclinical rejection. All samples were taken in the first year after transplantation, and the majority in the first 3 months after transplantation, reflecting the real-life timing of the majority of indication biopsies but obviating generalization of the results to biopsies later after transplantation and chronic rejection phenotypes. Induction therapy was different between the two clusters, with more induction therapy given to sensitized patients, and could have confounded cytokine levels early after transplantation. However, we would expect the induction therapy to cause a lesser immune activation, whereas here the opposite was observed. T cell sensitization was not measured, and its effect could therefore not be explored in this study. Also, we used a preselected multiplex assay of cytokines, chemokines, and growth factors, but other potentially important proteins were not studied. Downstream analyses were confined to the main clinical association, namely, DSA-mediated injury. This implies that other rejection types that could also contribute to cytokine expression and secretion were not studied in the downstream analyses. Nevertheless, we did not find indications for their contribution in the clinical associations. With regard to the *in vitro* analysis, we chose to focus on NK cells and non-classical monocytes, while other cell types capable of antibody recognition like CD16+ CD8 T cells (69), NKT cells, γδ T cells, neutrophils, and macrophages could also play a role in the chemokine profiles associated with DSA. Moreover, we did not study the effect of complement, which can cause endothelial injury from antibodies independent of immune cells (70). We also did not study the effect of missing self, which could enhance NK cell activation in addition to DSA (43, 71). Finally, the *in vitro* experiments in this study are not adequate to define the exact cellular source of the secreted cytokines.

This study did not intend to explore the biomarker potential of these cytokines. Although biomarker potential is not excluded, different study design and analyses would be needed to assess this. Furthermore, one could question the value of peripheral blood cytokines as clinical biomarkers as we can easily measure the gold standard, which are the circulating HLA-DSA.

In conclusion, this study demonstrates that blood pro-inflammatory cytokines are increased in kidney transplant patients with HLA-DSA and associate with worse graft survival, even in the absence of histology of rejection. These observations illustrate that HLA-DSA can lead to immune activation that is not always reflected in the histology of the concomitant kidney biopsy, challenging the concept that histology is the gold standard for identification of ongoing allo-immune activation after transplantation.

## Data Availability Statement

All of the individual de-identified participant data that underlie the results reported in this article (text, tables, figures, and appendices) can be made available on a collaborative basis following institutional review board approval. Proposals should be directed at the corresponding author.

## Ethics Statement

The studies involving human participants were reviewed and approved by the local ethical committee (S53364 and S61971). The patients/participants provided their written informed consent to participate in this study.

## Author Contributions

EVL and MN conceived and designed the study. EVL, MC, HdL, M-PE, PK, DK, AS, BS, AVC, and MN collected the clinical data and samples. SC performed the Luminex analyses. EVL and BL performed the scRNASeq analyses. TB, OT, and BL performed the *in vitro* experiments. EVL, BL, and MN did the statistical analyses and interpreted the data. EVL and MN wrote the draft of the report. All authors revised the report. All authors contributed to the article and approved the submitted version.

## Funding

EVL holds a fellowship grant (1143919N) from The Research Foundation Flanders (F.W.O.). MN is a senior clinical investigator of F.W.O. (1844019N) and is supported by an F.W.O TBM grant (grant no. T004417N), an F.W.O. junior project grant (grant no. G087620N), an ERACoSysMed H2020 grant (grant no. JTC2_29), and a C3 internal grant from the KU Leuven (grant no. C32/17/049).

## Conflict of Interest

The authors declare that the research was conducted in the absence of any commercial or financial relationships that could be construed as a potential conflict of interest.

## Publisher’s Note

All claims expressed in this article are solely those of the authors and do not necessarily represent those of their affiliated organizations, or those of the publisher, the editors and the reviewers. Any product that may be evaluated in this article, or claim that may be made by its manufacturer, is not guaranteed or endorsed by the publisher.

## Abbreviations

ABMR, antibody-mediated rejection; APC, antigen-presenting cells; DSA, donor-specific antibodies; ECa, activated endothelial cells; FcγR, Fc-gamma receptor; FDR, false discovery rate; HLA, human leukocyte antigen; NK, natural killer; PCA, principal component analysis; scRNASeq, single-cell RNA sequencing; TCMR, T cell-mediated rejection.
