# Supplementary material for: Circulating Donor-Specific Anti-HLA Antibodies Associate With Immune Activation Independent of Kidney Transplant Histopathological Findings
Source: Front Immunol. 2022 Feb 23;13:818569. doi: 10.3389/fimmu.2022.818569 (PMC8904423; doi:10.3389/fimmu.2022.818569)

**Supplementary Appendix**

**Circulating donor-specific anti-HLA antibodies associate with immune activation independent of kidney transplant histopathological findings**

Elisabet Van Loon^1,2^, Baptiste Lamarthée^1^, Thomas Barba^3^, Sandra Claes^4^, Maarten Coemans^1,5^, Henriette de Loor^1^, Marie-Paule Emonds^1,6^, Priyanka Koshy^7^, Dirk Kuypers^1,2^, Paul Proost^8^, Aleksandar Senev^1,6^, Ben Sprangers^1,2,8^, Claire Tinel^1^, Olivier Thaunat^3^, Amaryllis Van Craenenbroeck^1,2^, Dominique Schols^4^, Maarten Naesens^1,2^

**Table of Content**

- **Supplementary Methods**
- **Suppl. Table S1.** Ranges and percentages of values below the detection limit of the 28 cytokines**.**
- **Suppl. Table S2**. Demographics of the study population (N=192).
- **Suppl. Table S3**. Specificity and strength of the HLA-DSA at time of a first biopsy in cluster I vs. II.
- **Suppl. Fig. S1.** PCA and heatmap analyses in all biopsies (N=293).
- **Suppl.** **Fig. S2**. Distribution of cytokines in relation to detection limit in the two clusters.
- **Suppl. Fig. S3.** Forest plots of Odds ratio’s for association of the cytokines with rejection subtypes.
- **Suppl. Fig. S4.** Odds ratio’s and FDR corrected p-values resulted from logistic mixed models in all samples for cytokines and histological lesions of acute rejection.
- **Suppl. Fig. S5.** Analytical pipeline for the single-cell RNAseq analysis.
- **Suppl. Fig. S6.**  Distribution of cytokines in the *in vitro* NK cell experiment supernatants for DSA and isotype antibody condition.
- **Suppl. Fig. S7.** Distribution of cytokines in the *in vitro* NK cell and monocyte experiment supernatants for DSA and isotype antibody condition.
- **Suppl. Fig. S8.** Comparison of cytokines in the supernatants of GENC with vs. without DSA.
- **Suppl. Fig. S9.** Comparison of cytokine levels in supernatants of immune cells alone vs. with addition of GENC.

**Supplementary Methods**

*Study design and patient population*

Serum samples were collected at time of a concomitant kidney transplant biopsy. In total, we included 293 samples from 192 individual transplantations, each with a concomitant indication biopsy of adequate quality. Of the 192 patients, 117 patients had one biopsy and one concomitant blood sample, while 75 patients had multiple peripheral blood samples, each with a concomitant adequate biopsy sample. Polyoma and cytomegalovirus viremia were determined positive when above detection limit within thirty days of the index sample, negative when below the detection limit and missing when no value was available within thirty days.

*Clinical pathology*

Borderline changes were diagnosed as foci of tubulitis (t > 0) with minor interstitial inflammation (i1) or moderate-severe interstitial inflammation (i2 or i3) with mild (t1) tubulitis. We identified histology of ABMR (ABMR_h_) by positivity for the two first Banff criteria for active ABMR according to the Banff 2019 classification^1^, not taking into account donor-specific antibodies or gene expression changes. No rejection (NR) was defined as absence of ABMR, TCMR, and or borderline changes. Mixed rejection was defined as ABMR concomitant with TCMR or borderline changes. The chronic TCMR phenotype was not considered in this study. We considered a composite score of glomerulitis (Banff g) and peritubular capillaritis (Banff ptc) ≥2 as microvascular inflammation and a composite score of tubulitis (Banff t) and interstitial inflammation (Banff i) ≥2 as tubulo-interstitial inflammation. A humoral lesion score was defined as the sum of the scores of glomerulitis, peritubular capillaritis, intimal arteritis, thrombi and C4d deposition in the peritubular capillaries. A cellular lesion score was defined as the sum of tubulitis, interstitial inflammation and intimal arteritis scores.

*Detection of circulating DSA*

A possible presence of HLA-DSA was suspected at background-corrected mean fluorescence index (MFI) value around 500. However, for the final assignment of HLA-DSA the sera reactivity of the patients was analyzed. For most patients, anti-HLA donor-specific antibodies (HLA-DSA) were assessed retrospectively taking into account both donor and recipients high-resolution HLA genotyping results. All transplantations were performed with compatible complement-dependent cytotoxicity crossmatches on T and B cells. Pretransplant HLA-DSA were considered positive when HLA-DSA were present at time of transplantation. HLA-DSA were considered *de novo* when HLA-DSA arose after transplantation, up till the time the biopsy was taken.

*Cytokine analysis*

Peripheral blood serum samples were collected on the day of the concomitant indication biopsy in serum-separating tubes (BD Vacutainer®), centrifuged at 1900*g* for 10 minutes, 24°C and stored at -80°C. Expression of 27 cytokines, chemokines and growth factors was assessed using a 27-multiplex analysis following manufacturer’s instructions (BIORAD, M50-0KCAF0Y; Temse, Belgium) using Bio-Plex Multiplex immunoassay using Luminex magnetic beads. Levels of IL-1β,IL-1Ra, IL-2, IL-4, IL-5, IL-6, IL-7, CXCL8/IL-8, IL-9, IL-10, IL-12p70, IL-13, IL-15, IL-17α, CCL11/Eotaxin, basic-FGF, G-CSF, GM-CSF, interferon(IFN)-γ, CXCL10/IP10, CCL2/MCP-1, CCL3/MIP1α, CCL4/MIP1β, PDGF-BB, CCL5/RANTES, tumor necrosis factor(TNF)-α, and VEGF were determined. Additionally, a singleplex Bio-Plex analysis for CXCL9/MIG was performed following manufacturer’s instructions (BIORAD, Nazareth, Belgium). The samples were divided on separate 96-well plates. Standard curves were generated, and the cytokine concentrations were estimated with the Bio-Plex Manager software version 6.2 (Bio-Rad Laboratories, Hercules, California) using the five-parameter logistic model. When a value under the detection limit was recorded, half of the detection limit was imputed for analysis. Percentages of values below the detection limit for each protein are represented in **Supplementary Table S1.**

*Single cell RNAseq analysis*

Raw gene expression matrices generated per sample were merged and analyzed using Seurat V4 package^2^. Seurat objects were created and filtered with the following parameters: cells having <500 genes detected and having greater than 25% mitochondrial transcripts were excluded. After filtering, all objects were integrated using the SCTransform integration workflow on Seurat^3^. Briefly, 3000 features were used for integration using PrepSCTIntegration function and FindIntegrationAnchors function was used to limit batch effect. A full data set UMAP was generated using Seurat’s DimPlot function using the top 16 principal components. Clusters were built using the FindNeighbors and FindClusters functions in Seurat (resolution = 0.6). Cluster identification was performed using FeaturePlot function by evaluating the expression of specific markers in each cluster. Dot plots, violin plots and heatmaps were generated using the DotPlot, VlnPlot and DoHeatmap plotting function respectively in Seurat, with normalized counts in the RNA assay as input data.

*NK cell and monocyte sorting*

Peripheral blood mononuclear cells (PBMCs) were isolated from the blood of healthy volunteers from the Etablissement Français du Sang (Lyon, France) or from the Belgian Red Cross Flanders by Ficoll gradient centrifugation (Eurobio, Courtaboeuf, France). NK cells were purified from PBMCs by negative selection with magnetic enrichment kit (Stemcell, Grenoble, France) and non-classical CD16+ MDC8+ monocytes were isolated from PBMCs using Slan-(M-DC8)+ Monocyte Isolation Kit, (Miltenyi Biotec)^4^. All sorted cell populations exhibited high purity (>90%), as revealed by flow cytometry.

*CiGENC– NK cell coculture*

The human conditionally immortalized glomerular endothelial cell line^5^(ciGENC; HLA-A2) was purchased from University of Bristol. ciGENC were cultured in endothelial cell growth medium 2 (PromoCell, cat # C-22211) at 33°C in 5% CO2 for maintenance and were put at 37°C in 5% CO2 for 7 days to allow differentiation before being used for NK cell activation experiments. ciGENC (5.10^4^ cells) were seeded in flat-bottomed 96-well plates and let to adhere overnight at 37°C. When indicated, the cells were then incubated with either anti-HLA-A2 DSA containing serum or control human antibody serum during 30’ at room temperature before being washed with phosphate buffered saline. The anti-HLA A2 serum was derived from a transplant recipient with anti-HLA A2 antibodies with MFI > 10.000 on Luminex. The control serum was a pool of sera derived from never-transfused male donors of AB blood group that tested negative for anti-HLA by Luminex. Purified NK cells were then mixed with ciGENC at a ratio of 1:1, centrifuged at 100*g* for 1 min, and incubated at 37°C at 5% CO2. After 4 hours of co-culture, supernatants were collected, and stored at -80°C, until analysis. Expression of 27 cytokines, chemokines and growth factors was assessed using the same 27-multiplex analysis as used for the clinical samples, following manufacturer’s instructions (BIORAD, M50-0KCAF0Y; Biorad, Nazareth, Belgium).

*GENC– NK–Monocyte coculture*

Glomerular endothelial cells (GENC, Cell System, US) were cultured in endothelial cell growth medium 2 (PromoCell, cat # C-22211) at 37°C in 5% CO2 until 80% confluence before being used for coculture. GENC (5.10^4^ cells) were seeded in flat-bottomed 96-well plates and let to adhere overnight at 37°C. When indicated, the cells were then incubated with either anti-HLA-A, -B, -C purified antibody (BD Biosciences, cat # 560187) or control isotype (BD Biosciences, cat # 553447) during 30’ at room temperature before being washed with phosphate buffered saline. Purified monocytes and/or NK cells were then mixed with GENC at a ratio of 1:1, centrifuged at 100*g* for 1 min, and incubated at 37°C at 5% CO2. After 24 hours of co-culture, supernatants were collected, and stored at -80°C, until analysis. Expression of 27 cytokines, chemokines and growth factors was assessed using the same 27-multiplex analysis as used for the clinical samples, following manufacturer’s instructions (BIORAD, M50-0KCAF0Y; Biorad, Nazareth, Belgium).

Given the different anticipated response time in NK cells and monocytes, we chose to study NK cell supernatants after 4 hours of co-culture, and monocytes supernatants after 24 hours of co-culture

**References**

1. Loupy A, Haas M, Roufosse C*, et al.* The Banff 2019 Kidney Meeting Report (I): Updates on and clarification of criteria for T cell- and antibody-mediated rejection. *Am J Transplant* 2020.

2. Hao Y, Hao S, Andersen-Nissen E*, et al.* Integrated analysis of multimodal single-cell data. *bioRxiv* 2020**:** 2020.2010.2012.335331.

3. Hafemeister C, Satija R. Normalization and variance stabilization of single-cell RNA-seq data using regularized negative binomial regression. *Genome Biol* 2019; **20:** 296.

4. Hofer TP, van de Loosdrecht AA, Stahl-Hennig C*, et al.* 6-Sulfo LacNAc (Slan) as a Marker for Non-classical Monocytes. *Front Immunol* 2019; **10:** 2052.

5. Satchell SC, Tasman CH, Singh A*, et al.* Conditionally immortalized human glomerular endothelial cells expressing fenestrations in response to VEGF. *Kidney Int* 2006; **69:** 1633-1640.

| Supplementary Table S1. Median (IQR) and percentage below detection limit of multiplex analysis for each protein in N=293 peripheral blood samples. For values below detection limit, half of the detection limit was used for analyses. | | | | | | |
| --- | --- | --- | --- | --- | --- | --- |
| *Cytokines* | *Gene symbol* |  | **Median**  **(interquartile range)**  **pg/mL** | **% of values below detection limit** | **% of values below detection limit**  **Cluster I** | **% of values below detection limit**  **Cluster II** |
| IFN-γ | *IFNG* | Interferon-γ | 0.55 (0.55 - 0.55) | 77.8% | 0% | 88.7% |
| TNF-α | *TNF* | Tumor necrosis factor α | 2.10 (2.10 - 6.82) | 53.9% | 0% | 61.5% |
| IL-1β | *IL1B* | Interleukin-1β | 0.12 (0.12 – 0.12) | 88.4% | 61.1% | 92.2% |
| IL-1Ra | *IL1RN* | Interleukin-1 receptor antagonist | 50.04 (29.76 – 85.34) | 5.8% | 38.9% | 1.2% |
| IL-2 | *IL2* | Interleukin-2 | 0.75 (0.75 – 1.88) | 62.1% | 25.0% | 67.3% |
| IL-4 | *IL4* | Interleukin-4 | 1.66 (1.05 – 2.31) | 0.7% | 0% | 0.8% |
| IL-5 | *IL5* | Interleukin-5 | 2.10 (2.10 – 2.10) | 76.8% | 94.4% | 74.3% |
| IL-6 | *IL6* | Interleukin-6 | 0.34 (0.34 – 0.34) | 92.8% | 47.2% | 99.2% |
| IL-7 | *IL7* | Interleukin-7 | 9.46 (6.09 – 13.05) | 11.3% | 83.3% | 1.2% |
| IL-9 | *IL9* | Interleukin-9 | 7.57 (5.62 – 10.30) | 2.0% | 0% | 2.3% |
| IL-10 | *IL10* | Interleukin-10 | 0.83 (0.83 – 2.74) | 57.3% | 69.4% | 55.6% |
| IL-12p70 | *IL12A* | Interleukin-12p70 | 0.83 (0.83 – 0.83) | 93.5% | 88.9% | 94.2% |
| IL-13 | *IL13* | Interleukin-13 | 0.67 (0.31 – 1.35) | 47.8% | 80.6% | 43.2% |
| IL-15 | *IL15* | Interleukin-15 | 5.15 (5.15 – 5.15) | 92.8% | 63.9% | 96.9% |
| IL-17α | *IL17A* | Interleukin-17α | 5.94 (3.65 – 9.07) | 12.6% | 0% | 14.4% |
| Chemokines |  |  |  |  |  |  |
| CXCL8/IL-8 | *IL8* | Interleukin-8 | 3.85 (2.37 – 6.30) | 9.2% | 11.1% | 8.9% |
| CXCL9/MIG | *CXCL9* | Monokine induced by gamma interferon | 307.92 (149.48 – 716.10) | 0.3% | 0% | 0.4% |
| CXCL10/IP-10 | *CXCL10* | Interferon inducible protein 10 | 157.83 (99.78 – 314.92) | 0% | 0% | 0% |
| CCL2/MCP1 | *CCL2* | Monocyte chemoattractant protein 1 | 20.48 (11.94 – 30.69) | 0% | 0% | 0% |
| CCL3/MIP-1α | *CCL3* | Macrophage inflammatory protein 1α | 2.70 (1.97 – 3.72) | 0% | 0% | 0% |
| CCL4/MIP-1β | *CCL4* | Macrophage inflammatory protein 1β | 14.44 (10.88 – 20.57) | 0% | 0% | 0% |
| CCL5/RANTES | *CCL5* | Regulated upon Activation, Normal T cell Expressed, and Secreted | 1318.66 (905.68 – 1691.69) | 0% | 0% | 0% |
| CCL11/Eotaxin | *CCL11* | Eotaxin | 77.77 (47.98 – 110.35) | 0.3% | 0% | 0.4% |
| Growth Factors |  |  |  |  |  |  |
| Basic-FGF | *FGF2* | Basic Fibroblast Growth Factor | 6.54 (5.24 – 8.86) | 3.0% | 0% | 3.5% |
| G-CSF | *CSF3* | Granulocyte Colony Stimulating Factor | 173.26 (103.90 – 238.36) | 7.2% | 58.3% | 0% |
| GM-CSF | *CSF2* | Granulocyte Macrophage Colony Stimulating Factor | 0.27 (0.27 – 0.27) | 88.7% | 38.9% | 95.7% |
| PDGF-BB | *PDGFB* | Platelet derived growth factor BB | 885.63 (526.59 – 1359.93) | 1.0% | 0% | 1.2% |
| VEGF | *VEGFA* | Vascular endothelial growth factor | 4.75 (4.75 – 4.75) | 78.8% | 86.1% | 77.8% |

| Supplementary Table S2. Patient characteristics (N=192). | |
| --- | --- |
|  | *Mean ± standard deviation*  *Or no (%)* |
| Recipient age (years) | 55.5 ± 11.6 |
| Donor age (years) | 53.6 ± 15.3 |
| Recipient sex (male) | 133 (69.3%) |
| Donor sex (male) * | 110 (58.2%) |
| Cold ischemia time (hours)* | 11.8 ± 5.5 |
| Type of donor |  |
| - Circulatory death | 42 (21.9%) |
| - Brain death | 139 (72.4%) |
| - Living | 11 (5.7%) |
| Repeat transplantation (yes) | 23 (12%) |
| Immunosuppressive regimen (TAC-MMF-CS) | 175 (91.2%) |
| Induction therapy | 66 (34.4%) |

* missing data: donor sex N=3; cold ischemia time N=3. TAC = tacrolimus; MMF = mycophenolate mofetil; CS = corticosteroids

| Supplementary Table S3. Specificity and MFI for patients with HLA-DSA at the time of their first biopsy. | | | | | |
| --- | --- | --- | --- | --- | --- |
| Cluster I | | | Cluster II | | |
| Patient | Specificity | MFI | Patient | Specificity | MFI |
| 1 | A1 / B35 | 440 / 1130 | 1 | DP3 | 6864 |
| 2 | DRB1*04:03 pretransplant – resolved at time of first indication biopsy | 4406 when tested 2 months prior to sample - negative at time of first indication biopsy | 2 | C17 | 2981 |
| 3 | B14 | 1380 | 3 | A1 /B57 | 650 / 2964 |
| 4 | B39 | 826 |  |  |  |
| 5 | DR7 | 4177 |  |  |  |
| 6 | DQ8 | 706 |  |  |  |
| 7 | DQ3 | 1280 |  |  |  |
| 8 | Cw5 | 12920 |  |  |  |
| 9 | DR15 | 800 |  |  |  |
| 10 | B63 / DR7 (*de novo)* | 8127 /5121 |  |  |  |
| 11 | DQ2 | 7725 |  |  |  |
| 12 | A24 / DP13 (84DEAV) | 250 / 320 (1430) |  |  |  |
| 13 | DRB1*14 (*de novo)* | 5262 |  |  |  |
| 14 | DQ6 | 1060 |  |  |  |
| 15 | DQ5 | unknown |  |  |  |

**Supplementary Figure S1.** PCA and heatmap analyses in all biopsies (N=293). **A.** Principal component analysis (PCA) based on the information from the 28 blood protein levels. Colours indicate the rejection phenotypes and symbols the presence of HLA-DSA. **B.** Heatmap analysis of histological lesions and blood proteins. Reordering of dendrograms based on hierarchical clustering. Column side colours indicate the HLA-DSA status, HLA-antibodies, cellular score, humoral score, rejection phenotype and whether the first biopsy of this patient belonged to cluster I from Figure 1 for each sample. ABMR= Antibody-mediated rejection; TCMR= T cell-mediated rejection; Mixed rejection was defined as ABMR concomitant with TCMR or borderline changes; HLA-DSA= anti-HLA donor-specific antibodies. All protein levels are log10 transformed and in pg/mL. Humoral score= sum of glomerulitis, peritubular capillaritis, intimal arteritis and C4d deposition in the peritubular capillaries. Cellular score = sum of tubulitis, interstitial inflammation and intimal arteritis.
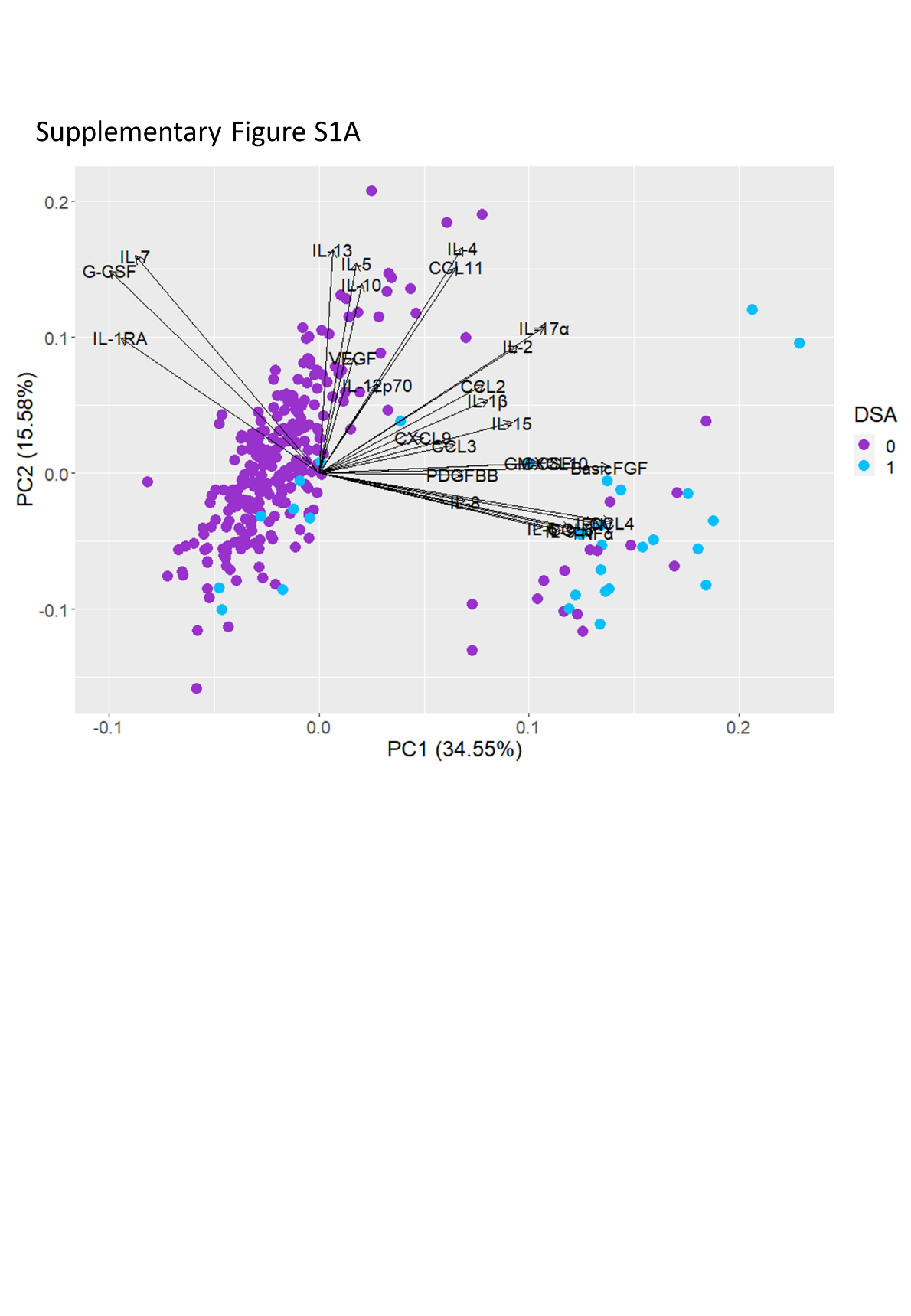


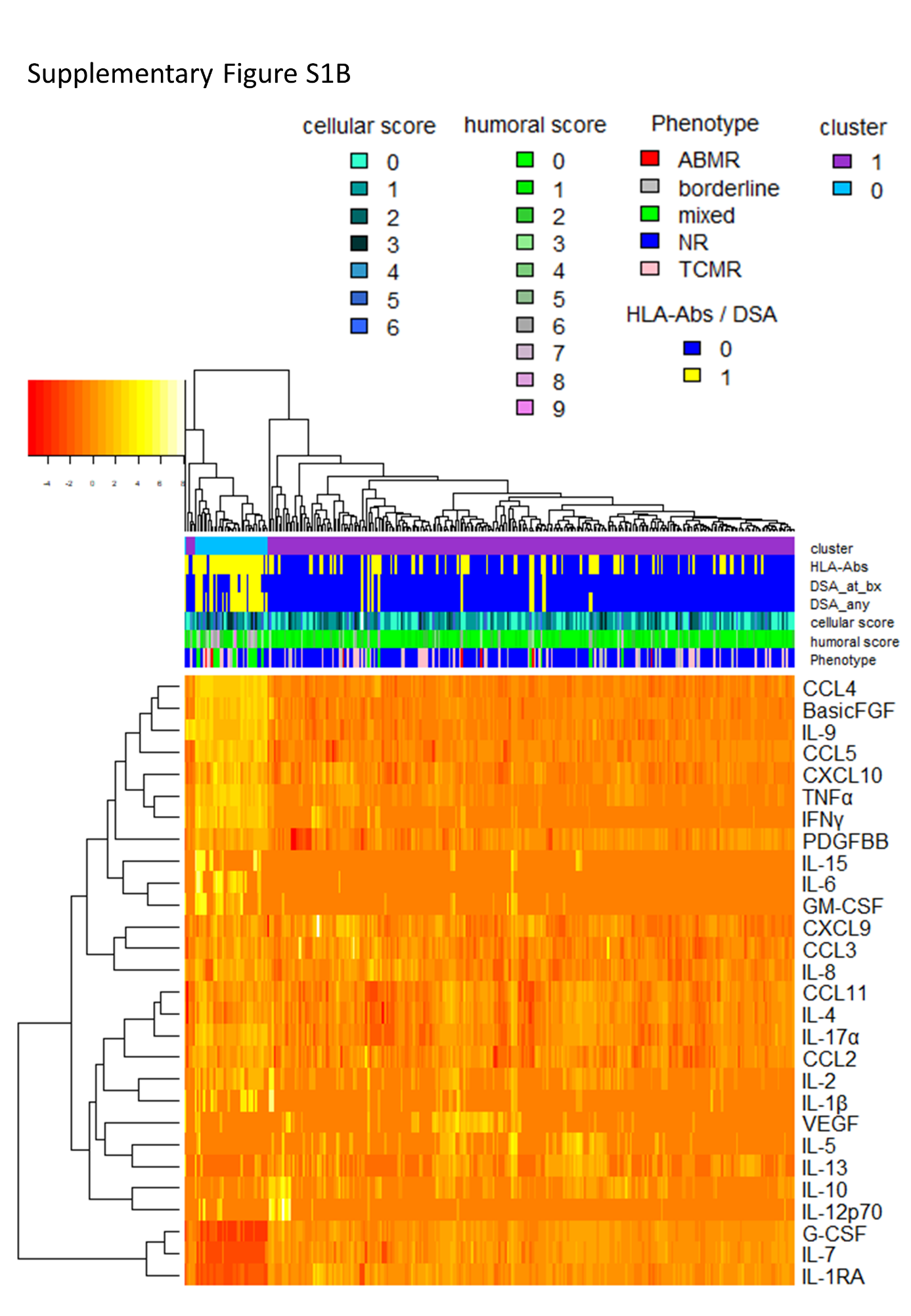


**Supplementary Figure S2. Distribution of cytokines in relation with detection limit for cluster I (N=20) and cluster II (N=172) in the first biopsies (N=192).**

**
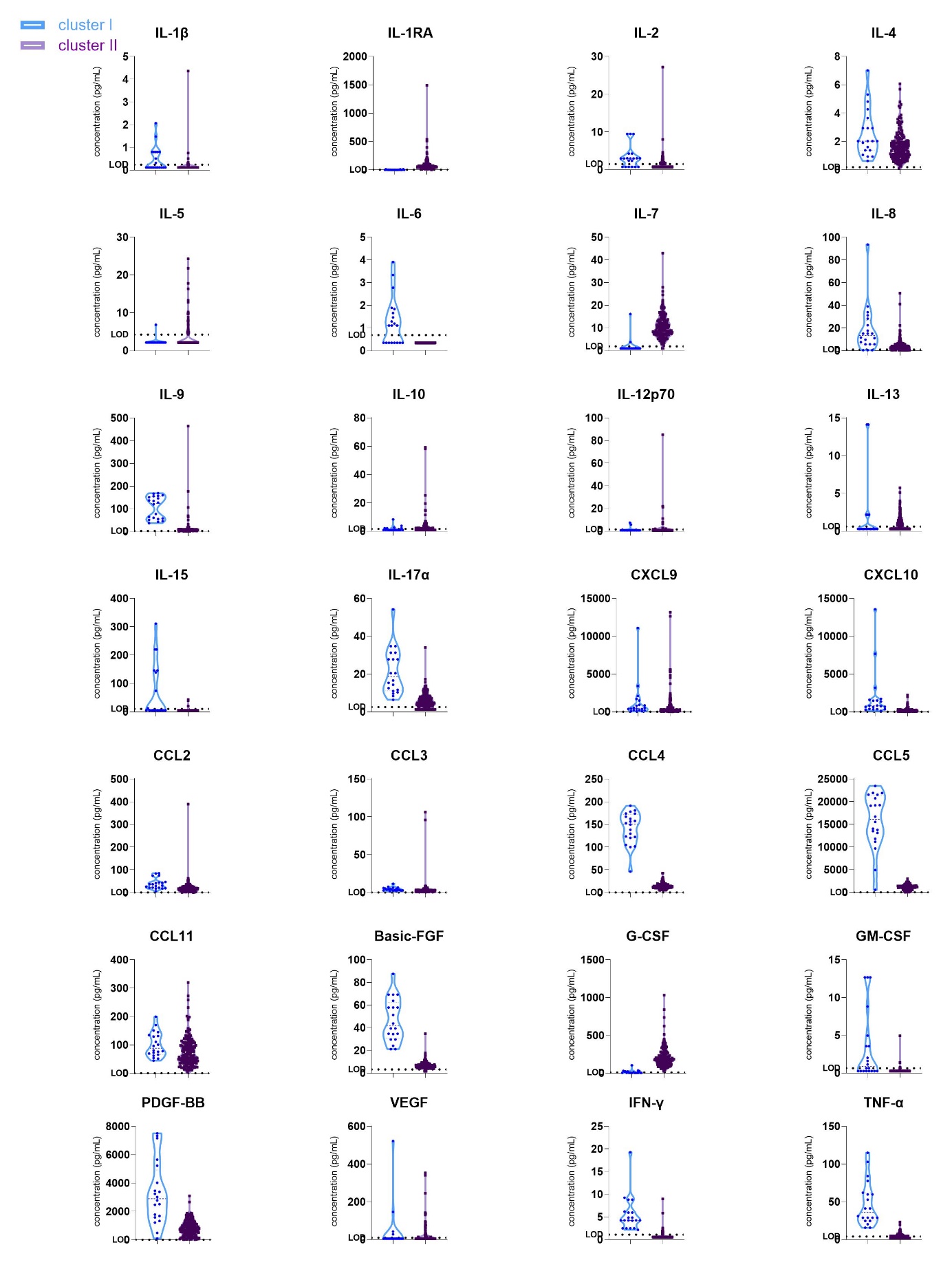
**

**Supplementary Figure S3**. Forest plots of Odds ratio’s for association of the cytokines with rejection subtypes. Panel A: Any rejection (N=106) vs. no rejection (N=187). Panel B: TCMR (N=71) vs. no TCMR (N=222). Panel D: borderline changes (N=28) vs. no rejection (N= 187). Blue color indicates associations that were significant after false discovery rate correction (FDR p-value <0.05). TCMR= T-cell mediated rejection; FDR= false discovery rate.


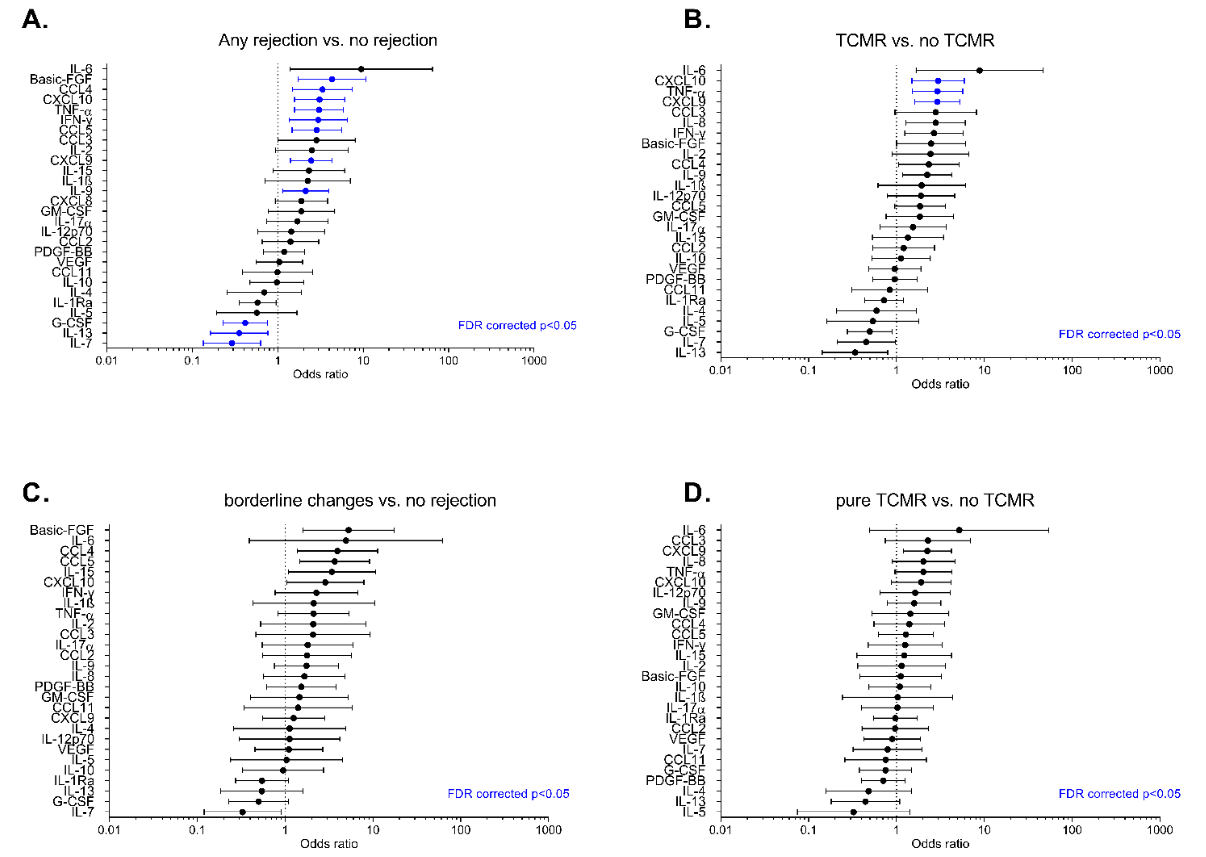


**Supplementary Figure S4.** Odds ratio’s and FDR corrected p-values resulted from logistic mixed models in all samples for 28 chemokines and histological lesions of acute rejection. Odds ratios are shown on the x axis, p values (-log10 transformed) on the y axis. P=0.05 is indicated by the horizontal dotted line (and FDR corrected). The vertical dotted line indicates Odds ratio of 1, with downregulation on the left and upregulation the right. Antibody-mediated rejection (ABMR) related lesions are microvascular inflammation (mvi) (glomerulitis + peritubular capillaritis, <2 or ≥2). T-cell mediated rejection (TCMR) related lesions are hallmarked by tubulo-interstitial score (tubulitis + interstitial inflammation). Intimal arteritis lesions (v>0) can be present in both ABMR and TCMR.


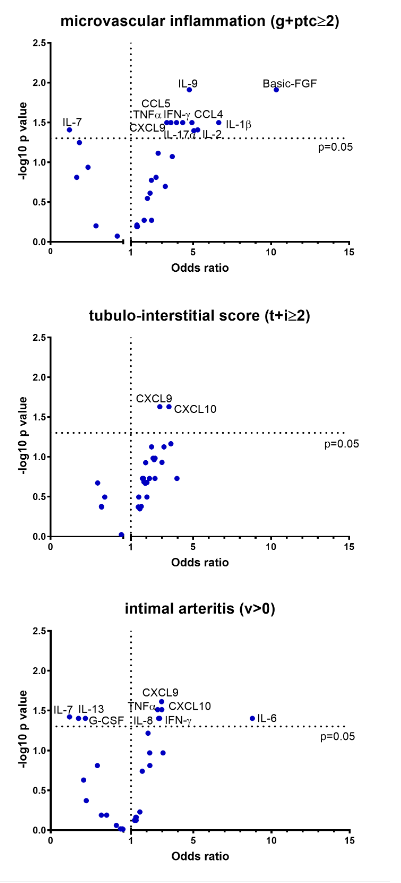


**Supplementary Figure S5. Overview of the analysis on publicly available single-cell data from kidney biopsies to analyze which cell types expressed the corresponding genes in the kidney.** Briefly, scRNAseq was performed on two renal allograft biopsies with the diagnosis of ABMR. ﻿These datasets were integrated with 5 healthy reference biopsies in order to seek for chemokine expressing cells. After quality control and filtering, 33216 cells were detected and unsupervised clustering revealed 14 clusters.

**
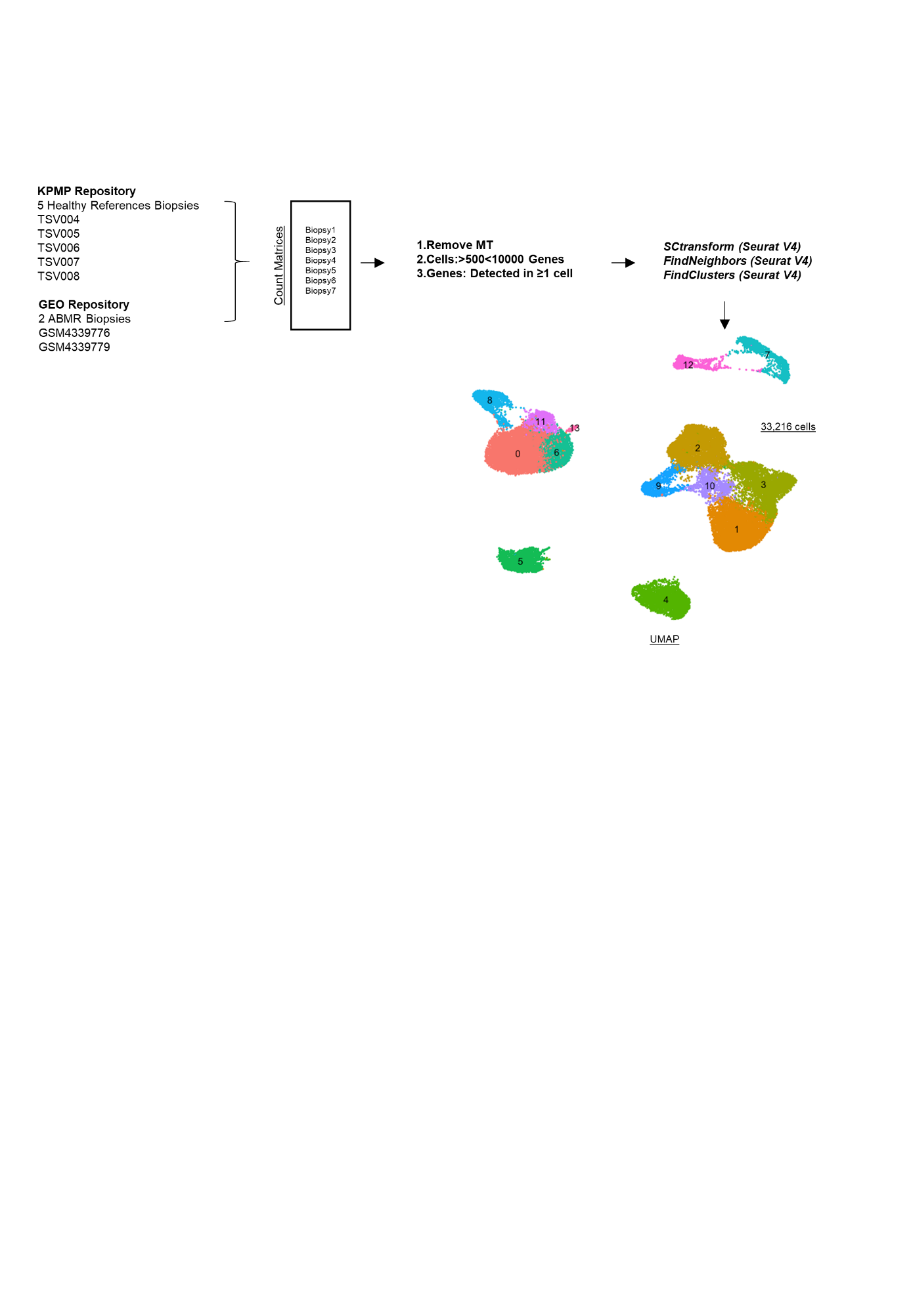
**

**Supplementary Figure S6.** NK cell experiment: comparison of DSA vs isotype for all cytokines. Levels of IL-7, IL-12p70, IL-13 and IL-17a were all below limit of detection and not shown.
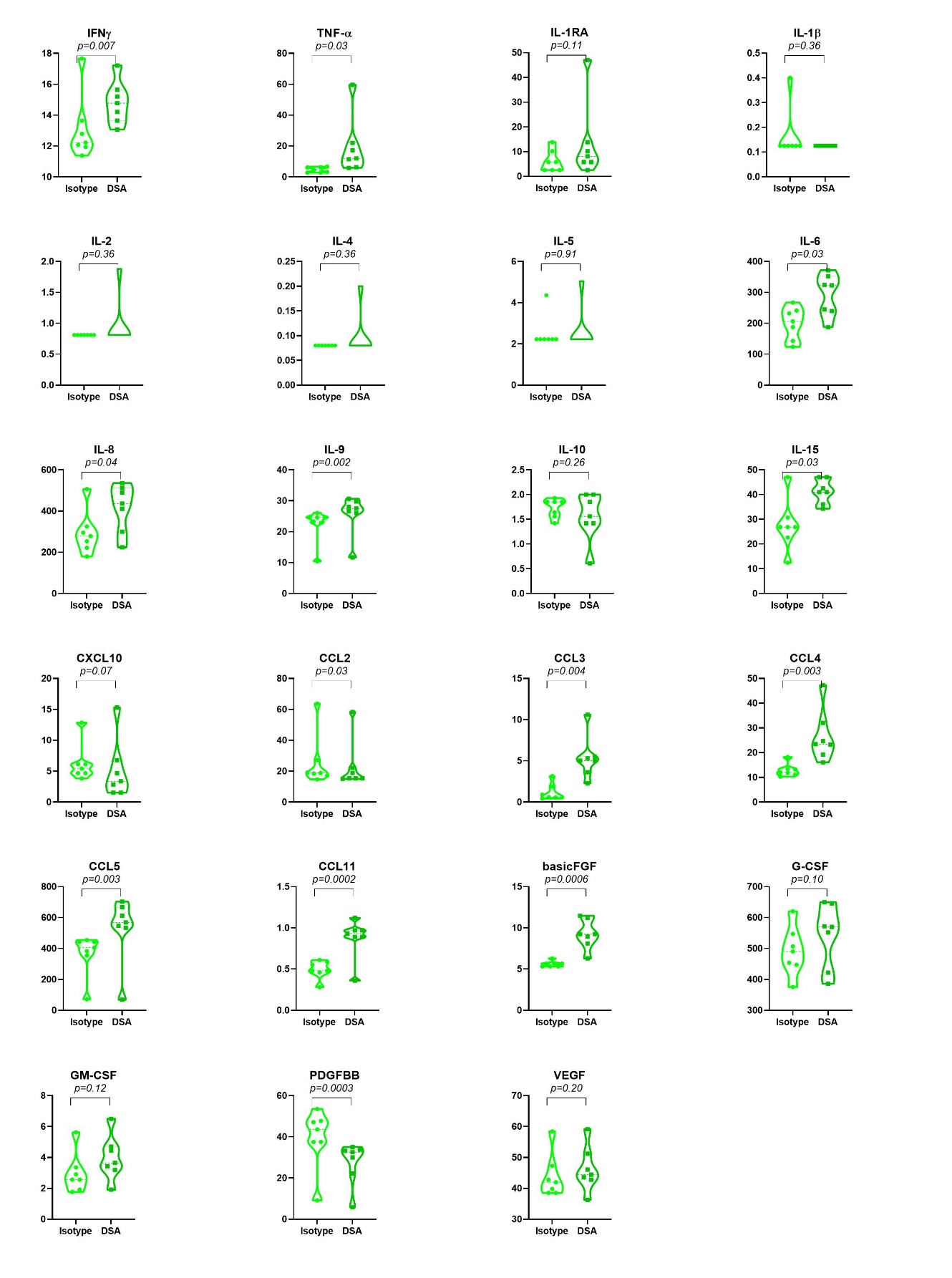


**Supplementary Figure S7**. NK + monocyte + GENC cell experiment: comparison of DSA vs. isotype for all cytokines. Levels of IL-7, IL-12p70 and IL-13 were all below limit of detection and not shown. GENC=primary glomerular endothelial cells.
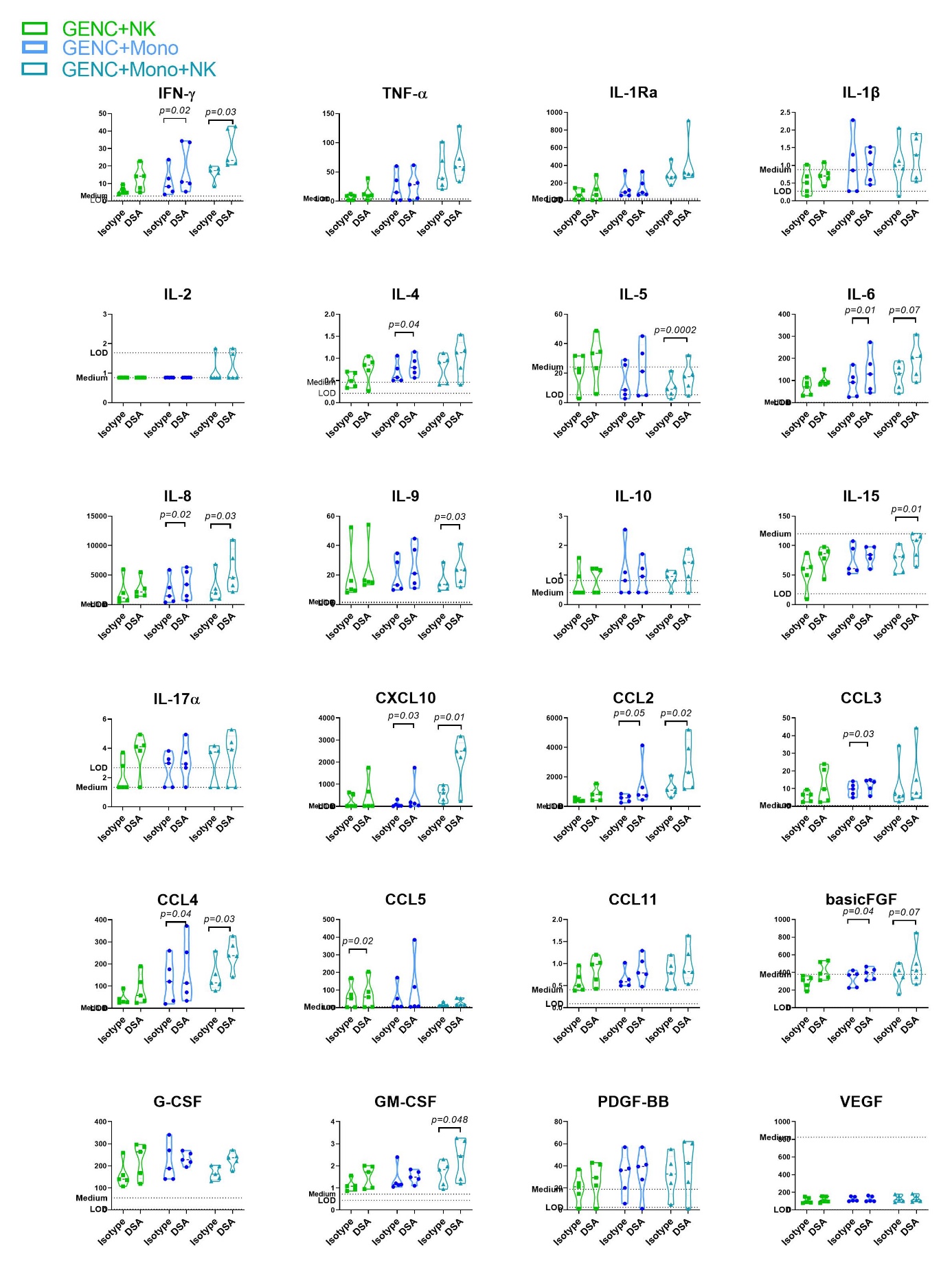


**Supplementary Figure S8. Comparison of DSA vs. isotype antibody in GENC alone after 24h of coculture.** Violin plots illustrating concentrations of the cytokines in the supernatants harvested after 24h of coculture of primary glomerular endothelial cells (GENC) with isotype antibody vs. HLA class I specific antibody (DSA). The limit of detection (LOD) concentration and the concentration in the medium alone are indicated. Comparison between the condition was done using T-tests of the log10 transformed data. Levels of IL-2, IL-7, IL-12p70, IL-13 were all below the limit of detection and therefore not shown.


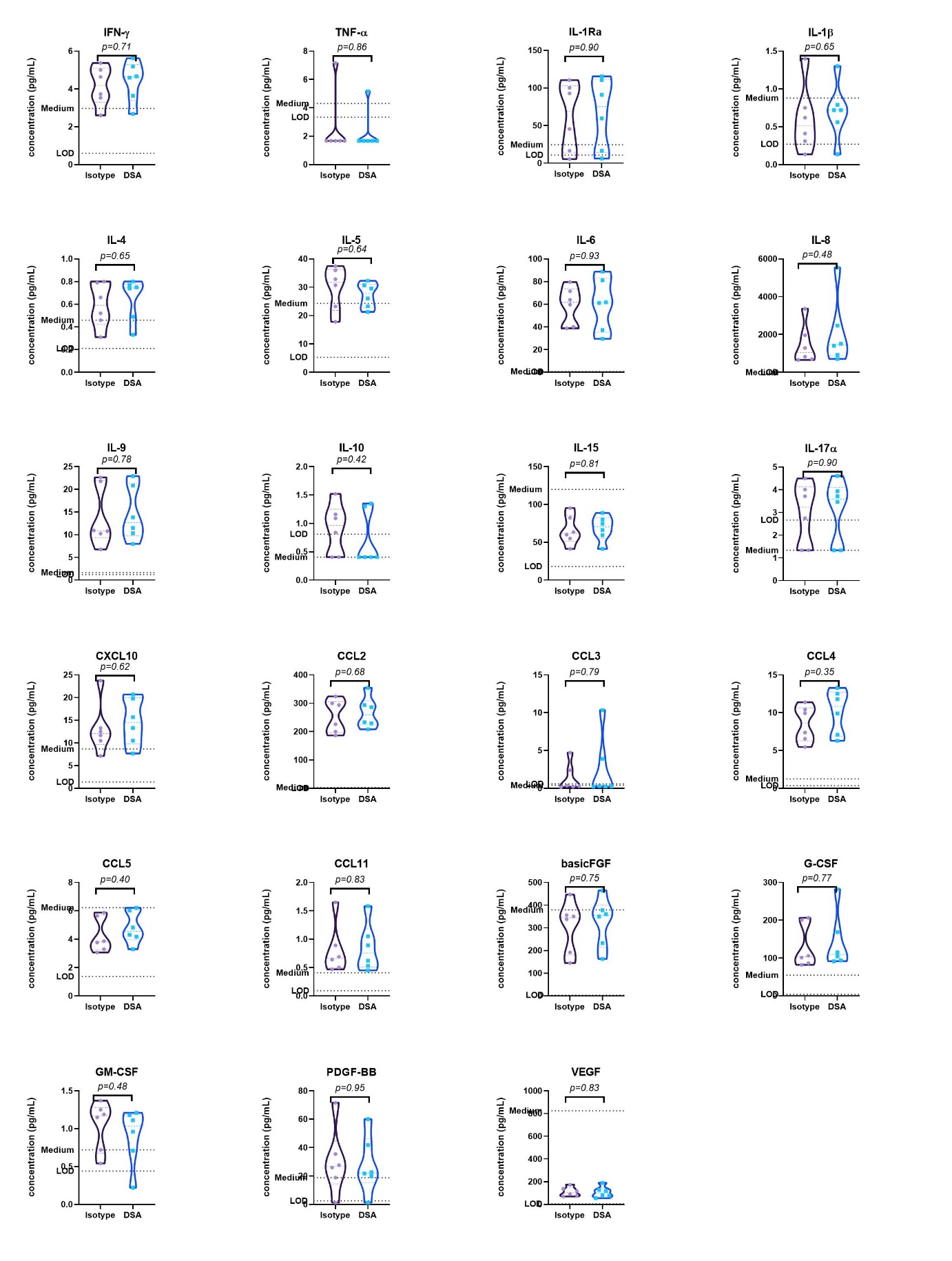


**Supplementary Figure S9. Comparison at 24h of supernatants of immune cells alone vs. immune cells + GENC.** For IL-7, IL-12p70 and IL-13 all values were below LOD, these data are not shown.


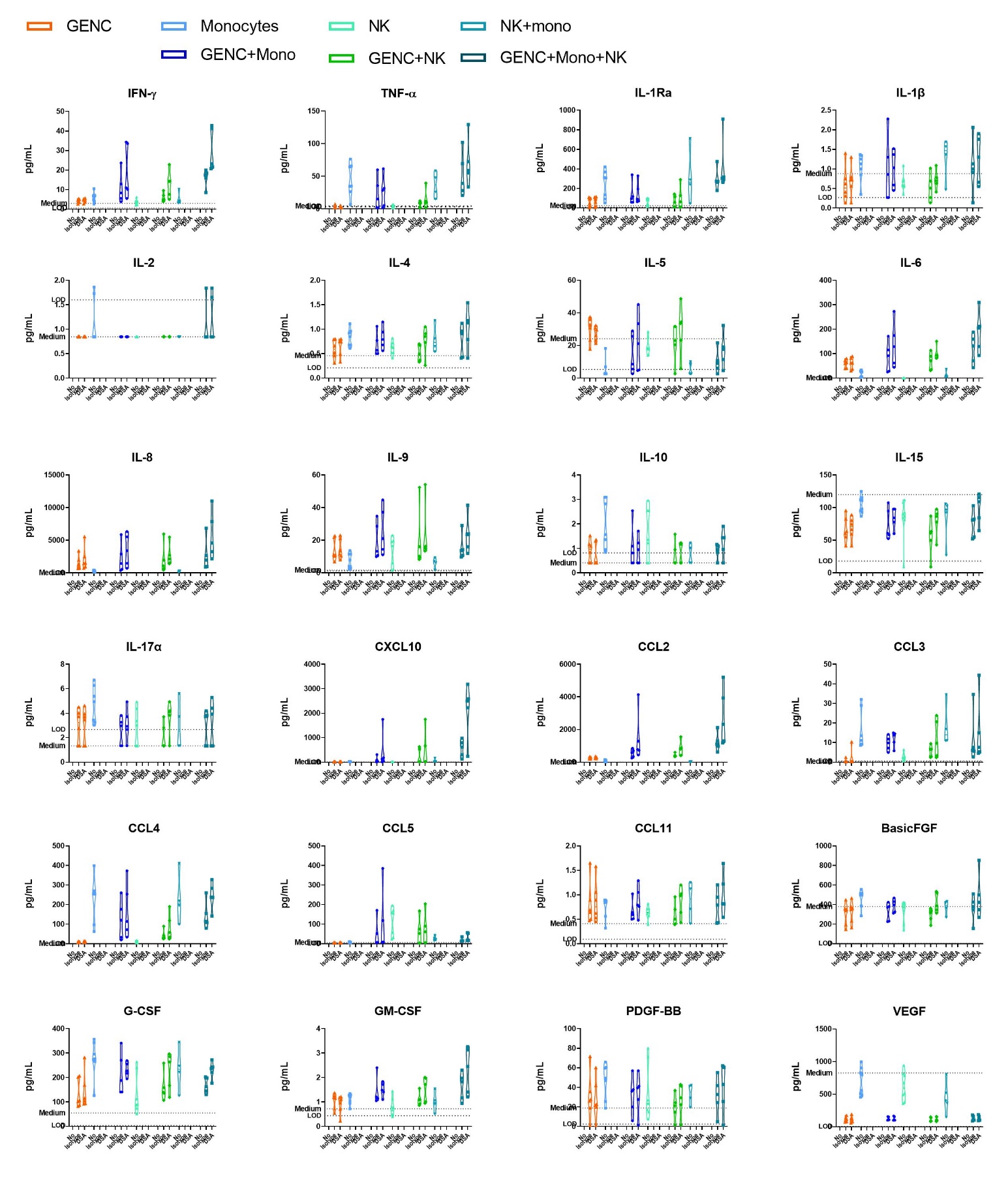

Supplement: Supplementary file 1 [file DataSheet_1.docx]
